# Molecular Mechanism of Gleditsiae Spina for the Treatment of High-Grade Serous Ovarian Cancer Based on Network Pharmacology and Pharmacological Experiments

**DOI:** 10.1155/2022/5988310

**Published:** 2022-03-08

**Authors:** Boran Zhang, Wenchao Dan, Ganlin Zhang, Xiaomin Wang

**Affiliations:** ^1^Beijing University of Chinese Medicine, Beijing, China; ^2^Oncology Department, Beijing Hospital of Traditional Chinese Medicine, Capital Medicine University, Beijing, China

## Abstract

**Background:**

Gleditsiae Spina, widely used in traditional Chinese medicine, has a good curative effect on malignant tumors such as ovarian cancer, but the mechanism is not clear. So, we aimed to analyze the pharmacological mechanism of Gleditsiae Spina in the treatment of high-grade serous ovarian cancer (HGSC) based on network pharmacology and biological experiments.

**Methods:**

The main active ingredients of Gleditsiae Spina were identified by high performance liquid chromatography (HPLC) and mass spectrometry (MS), and the active ingredients were performed by ADME screening. The component targets of Gleditsiae Spina were screened using the PharmMapper platform, and differentially expressed genes in normal and HGSC tissues were identified through the GEO database. Thereafter, the network of “active ingredient-targets” was constructed by cytoscape 3.7.2 software. The protein-protein interaction network was established by the BioGenet database to mine the potential protein function. Biological processes and pathways were analyzed through Gene Ontology and Kyoto Encyclopedia of Genes and Genomes analysis. The binding ability of the core components of the Gleditsiae Spina and the core target of HGSC was verified by molecular docking and molecular dynamics simulation, and the therapeutic effect of Gleditsiae Spina was proved in vitro through cytotoxicity experiments. The effect of Gleditsiae Spina on the core pathway is obtained by western blotting.

**Results:**

Gleditsiae Spina had cytotoxicity on HGSC based on network pharmacology and biological experiments. Luteolin, genistein, D-(+)-tryptophan, ursolic acid, and berberine are the identified core active ingredients of Gleditsiae Spina for regulating HGSC, with HPSE, PI3KCA, AKT1, and CTNNB1as the ideal targets. The prediction results were verified by molecular docking, molecular dynamic simulation, cell viability, and western blot analysis.

**Conclusion:**

Gleditsiae Spina mainly downregulates the expression of heparanase and *β*-catenin to affect the composition of tumor cytoplasmic matrix and can regulate the PI3K-AKT pathway, integrating multiple targets and multiple pathways to play a therapeutic role. It also provides a theoretical basis for the prevention of ovarian cancer and its treatment using traditional Chinese medicine in the future.

## 1. Introduction

As one of the most serious diseases in women, ovarian cancer causes 152 000 deaths per year worldwide, ranking fourth among female tumor deaths [1–3]. The majority of patients are diagnosed at the advanced stage of ovarian cancer due to the lack of obvious symptoms and reliable diagnostic tools [4]. Therefore, improving ovarian cancer treatment, particularly highly serous ovarian cancer (HGSC), has great clinical significance. As an aggressive cancer, HGSC often shows aneuploidy and mutations and is considered to be highly malignant, and their clinical prognosis is worse than other classifications [5]. Operative treatment includes total abdominal hysterectomy, bilateral salpingectomy, removal of pelvic and para-aortic lymph nodes, and omentum (such as appendectomy), combined with the usage of platinum drugs and taxane-like chemotherapy [6]. Traditional treatments such as cytoreductive surgery and platinum-based chemotherapy, may cause 75-80% of relapses [7], and the overall survival of HGSC patients has not been noteworthy in the past 30 years.

Currently, traditional Chinese medicine (TCM) is being increasingly recognized due to its effectiveness in alleviating illnesses with minimal side effects [8]. The application of TCM in the treatment of tumors is increasing extensively in China and most Asian countries in the past decades. TCM plays an important role in the prevention and treatment of precancerous lesions, postoperative recurrence of tumors, reduction of toxic side effects of western medicine, and maintenance of advanced tumors [9]. In a previous meta-analysis, the combination of TCM treatments and western medicine significantly improved the KPS score, CA125 level, and 3-year survival rate in the postoperative adjuvant treatment of ovarian cancer [10]. The Chinese herbal medicine “Shenlinglan Capsule” can attenuate ovarian cancer migration by inhibiting the expression of glycogen synthase kinase 3 (GSK-3) in vitro [11]. Studies have shown that “Jianpi Huayu Decoction” can alleviate the progression of liver cancer by reducing the expression of reactive oxygen species (ROS) in myeloid-derived suppressor cells (MDSCs), attenuating inhibition of CD4 cell proliferation [12]. Bufalin in the TCM “Chan su” can regulate the cell cycle and inhibit receptor phosphorylation and tumor cell proliferation [13].

Gleditsiate Spina, as a widely used Chinese medicine, with its extensive activities has been proved, including detoxification, reduction of swelling and purulence, wound healing, and bloated dystocia [14]. Meanwhile, it is an alternative remedy in the treatment of ovarian cancer clinically. HPLC elucidated various flavonoids present in Gleditsiae Spina extracts, which confers anti-inflammatory, antibacterial, and antitumor effects [15]. It is reported that the ethanol extracts of Gleditsiae Spina can maintain tumor cells proliferation in G2/M phase, regulate phosphorylation of extracellular signal-regulated kinases (ERK), tumor necrosis factor-alpha (TNF-*α*), matrix metalloproteinase-9 (MMP-9) expression, and to inhibit tumor cell growth [16]. However, the main biologically active ingredients and the molecular mechanism of antiovarian cancer are not yet be elucidated.

With the development of network technology and bioinformatics, network pharmacology is gradually becoming a novel tool in elucidating molecular mechanisms and pharmacological effects [17]. It can effectively establish a “compound-protein/gene-disease” network and reveal the mechanism of small molecules through high-throughput methods [18]. Furthermore, networks can be constructed between various components to analyze their mutual relationships. Careful investigation of the key nodes in the network can systematically explain the material basis and mechanisms of Chinese medicines. So, this research predicts the mechanism of Gleditsiae Spina in the treatment of ovarian cancer by network pharmacology methods. Based on the results obtained, the experimental verification is carried out, revealing the mechanism of traditional Chinese medicine in the treatment of ovarian cancer.

Therefore, in this study, we hope to reveal the scientific connotation of the treatment of ovarian cancer by Gleditsiae Spina and provide new support for the expansion of clinical research and the development of new drugs to improve clinical efficacy.

## 2. Methods

### 2.1. Preparation of Gleditsiae Spina

#### 2.1.1. Production of Freeze-Dried Powder

Gleditsiae Spina is administrated as medicine with dry spines of the leguminous Gleditsia sinensis Lam, and its Latin scientific name has been checked with the PlantList (http://www.theplantlist.org). In this study, Gleditsiae Spina was purchased as ready-to-use decoction pieces from Beijing Xinglin Pharmaceutical Co. (Beijing, China) and was identified by Professor Mao Ke-Chen according to the Chinese Pharmacopoeia standards.

Gleditsiae Spina decoction (450 mL) was prepared from 200 g Gleditsiae Spina pieces after twicely boiled in 1000 mL of distilled water 1 h and filtered through 200 mesh, and then the decoction was transferred to a vacuum freeze dryer (Epsilon 2-4LSC; Martin Christ Gefriertrocknungsanlagen, Harz, Germany) to obtain 18.72 g of freeze-dried powder, with a production rate of 9.36%. The powder was placed into a closed container containing silica gel to keep it dry. For subsequent experiments, the powder was dissolved in deionized water (100 mg/mL) and stored at −20°C.

#### 2.1.2. Ingredient Identification Using High Performance Liquid Chromatography and Mass Spectrometry

Gleditsiae Spina freeze-dried powder (100 mg) was dissolved in 50% methanol and sonicated for 10 min. The solution was centrifuged at 3,000 r/min for 5 min (Beckman Coulter, Brea, CA, USA), and the supernatant was collected and filtered through a 0.22 *μ*m filter (Millipore, Burlington, MA, USA). The filtered solution was subjected to mass spectrometry analysis using a Q-Exactive Orbitrap quadrupole-electrostatic field orbitrap mass spectrometer equipped with a thermal spray ion source and a Vanquish ultra-high performance liquid chromatography system (Thermo Fisher Scientific, Waltham, MA, USA).

### 2.2. Target Prediction

#### 2.2.1. Prediction of Active Components in Gleditsiae Spina

Based on the mass spectrometry results, the active ingredients of Gleditsiae Spina were initially selected. The molecular structure of the active pharmaceutical ingredients and 3Dsdf was obtained on the PubChem platform, the latter was uploaded to the SwissADME platform, and then the pharmacokinetics and drug similarity were screened. The compound ingredients were required to have good gastrointestinal absorption, and the drug similarity received more than 3 positive evaluations. The potential targets of the ingredients were predicted on the PharmMapper platform [19].We required the norm fit value to be greater than 0.75.

#### 2.2.2. Construction of the Active Ingredient-Target Network of the Gleditsiae Spina

The “active ingredient-target” network of the Gleditsiae Spina was constructed and analyzed by Cytoscape 3.7.2 software [20]. “Node” was used to indicate the component or target, and “edge” was used to indicate the relationship among them. The “Network Analyzer” analyzing tool built in Cytoscape 3.7.2 software was used to analyze the network characteristics, including degree, betweeness, and closeness, to study important components and target relationships of Gleditsiae Spina.

#### 2.2.3. Ovarian Cancer-Related Targets

The differentially expressed genes of patients with ovarian cancer were obtained from the GEO database (series: GSE54388 and GSE14407, samples: normal tissue GSM1314222-GSM1314227, GSM360039-360049, and GSM359984, and tumor tissue GSM1314228-GSM1314243 and GSM359972-359983). Differential genes with an adj. *P* value <0.05 and |log2(fold change)| > 1 were considered to be of significantly differential expression and ovarian cancer-related targets.

#### 2.2.4. Construction of Protein Interaction Network and Screening of Key Targets

The PPI were constructed by BisoGenet3.0 [21]. The targets were related to the active ingredients of Gleditsiae Spina, and the targets of disease were introduced into BisoGenet, each generated a PPI network. The intersection network of the two PPI networks was extracted through the merge function in Cytoscape, and CytoNCA2.1 [22] was used to analyze the nodes of the intersection network. The targets were mapped and visualized by Cytoscape 3.7.2, and the protein-protein internetwork (PPI) of the shared genes was constructed through the String APP in Cytoscape.

#### 2.2.5. Pathway Enrichment Analysis

The Metascape platform [23] was used to perform pathway enrichment analysis on the target. The platform integrates many authoritative functional databases such as GO and KEGG and supports batch genes or annotates, enriches and analyses proteins, and builds PPI networks. The platform is updated once a month to ensure data is reliable. Imported potential ovarian cancer targets were inserted into the Cytoscape platform for GO and KEGG analysis, and the results were saved and visualized with *R* software3.6.1.

#### 2.2.6. Molecular Docking and Molecular Dynamic Simulation

In order to further determine the credibility of the relationship between the ovarian cancer target and the core components of Gleditsiae Spina, the top two compounds of traditional Chinese medicine compound target were selected as ligands genistein and luteolin, and four important targets were selected to analyze molecularly docking.

First, the crystal structure of the three proteins in pdb format from the RCSB database was downloaded, and the SDF was from the PubChem database. We also downloaded the 3D chemical structure of the candidate compound and used Open Babel 2.4 to convert to the pdb format file. AutoDock Tools 1.5.6 was used to delete the water molecules in the ligand, separate the ligand from the receptor, add nonpolar hydrogen, calculate the Gasteiger charge, and save the pdbqt format file. The selected potential core constituent ligands were subjected to energy minimization treatment, the ligand atom type was given, and the charge was calculated and stored in pdbqt format. Molecular docking operations were performed using Autodock Vina 1.1.2 and reflect the matching degree and docking activity between the target and the ligand through the docking score value, where we believe that a docking score > 4.25 means that the ligand and the target have binding activity, a score > 5.0 means good matching activity, and a score > 7.0 means strong docking activity [24].

The MD simulation of docked complexes was carried out using Desmond version 2020. Here, OPLS3e force field was used to initiate the MD simulation, and the system was solvated using the TIP3 water model. The neutralization of the system was performed by adding counter ions. Energy minimization of the entire system was performed using OPLS3e, as it is an all-atom type force field. The geometry of water molecules, the bond lengths, and the bond angles of heavy atoms was restrained using the SHAKE algorithm. Simulation of the continuous system was executed by applying periodic boundary conditions, and long-range electrostatics was maintained by the particle mesh Ewald method. The equilibration of the system was done using NPT ensemble with temperature at 300 k and pressure at 1.0 bar. The coupling of temperature-pressure parameters was done using the Berendsen coupling algorithm. On postpreparation of the system, the production run was performed for 200 ns with a time step of 1.2 fs, and trajectory recording was done for every 200 ps summing up to the recording of 10,00 frames. The calculation of the RMSD (Root mean square deviation) was done for the backbone atoms and was analyzed graphically to understand the nature of protein-ligand interactions. RMSF (root mean square fluctuation) for every residue was calculated to understand the major conformational changes in the residues in comparison between the initial state and dynamics state.

### 2.3. Verification In Vitro

#### 2.3.1. Cells

Human ovarian cancer cell line A2780 purchased from ATCC was cultured in RPMI-1640 (Gibco Company, USA) with 10% foetal bovine serum (Gibco Company, USA) and 1% penicillin-streptomycin (10000 units/mL penicillin and 10000 *μ*g/mL streptomycin, Gibco Company, USA). Human cancer cell line SKOV3 and HEY purchased from ATCC were cultured in DMEM (Gibco Company, USA) and cultured at 37°C in a humidified atmosphere under 5% CO_2_. Human ovarian granulosa cell line SVOG and human ovarian epithelial cell line IOSE80 purchased from ATCC were cultured in RPMI-1640 (Gibco Company, USA) with foetal bovine serum and penicillin-streptomycin the same as the cell line A2780. Cells in the exponential growth phase were used in all experiments.

#### 2.3.2. Cell Proliferation

The MTT was used to evaluate cell proliferation and viability. 2000 cells were seeded in each of the nonedge well of 96-well plates. Freeze-dried powder solution of the Gleditsiae Spina was diluted by multiples, from 0.25 to 8 mg/mL and added after the cells adhered to the wall. 24 hours and 48 hours after Gleditsiae Spina treatment, 20 *μ*L of 3-(4,5-dimethylthiazol-2-yl)-2,5-diphenyltetrazolium bromide (MTT) (Sigma, St. Louis, MO, USA) solution (5 mg/mL in phosphate-buffered saline (PBS)) was added to the culture medium in each well at a final concentration of 5 *μ*g/mL, and the cells were incubated at 37°C for 4 hours. The supernatants were replaced with 150 *μ*L of dimethyl sulfoxide (Sigma, St. Louis, MO, USA). Then, the 96-well plates were measured by microplate reader at 490 nm.

#### 2.3.3. Western Blot

After exposure to the test compounds (1.25 mg/mL and 2.5 mg/mL freeze-dried powder of the Gleditsiae Spina) for 24 h, the A2780 cells were harvested and lysed with RIPA lysis buffer (Beyotime Biotechnology, Beijing, China) containing Protease Inhibitor Cocktail Set III (Calbiochem, San Diego, CA, USA). After centrifuged at 12,000 rpm for 15 min at 4°C, and the supernatants were collected, and the protein concentration was determined using the Pierce BCA Assay Kit (Thermo Scientific, Rockford, IL, USA). Equal amounts (30 *μ*g/lane) of total protein were separated by 10% SDS-PAGE and transferred onto PVDF membranes. The membranes were blocked with 5% BSA (Amresco, Solon, OH, USA) at room temperature for 1 h and incubated overnight at 4°C with the following primary antibodies: anti-HPSE1 (1 : 1000, CST,USA), anti-MMP9 (1 : 1000, CST,USA), anti-*β*-catenin (1 : 1000, CST, USA), anti-N-cad (1 : 1000 CST, USA), anti-E-cad (1 : 1000 CST, USA),anti-PI3K/p-PI3K (1 : 1000, CST,USA), anti-AKT/p-AKT (1 : 1000, CST,USA), anti-YAP/TAZ (1 : 1000, CST,USA) and *β*-actin (1 : 10000, CST,USA). After washing the membranes in Tris-buffered saline with 0.1% Tween-20 (TBST), the membranes were probed with secondary antibodies (1 : 10,000) for 1 h at room temperature. The signals were detected using an Odyssey Infrared Imaging System (Li-cor Biosciences, Lincoln, NE, USA). The relative density of the protein bands was measured by Odyssey version 3.0 software (LI-COR Biosciences). Each experiment was repeated three times. The ratios of the protein band intensities relative to that of *β*-actin were calculated for each sample using Image J.

#### 2.3.4. AKT Kinase Dependency Validation

AKT kinase inhibitor, purchased from MedChemExpress (HY-10249, MCE), was added to A2780 cells at the dose from 1 nM to 8 nM, and the expression of AKT in the cells with or without inhibitor was tested by WB. The appropriate inhibitor concentration (2 nM) was selected for the efficacy experiment. 3000 cells with AKT kinase inhibitor were seeded in each of the nonedge well of 96-well plates and after 24 hours, Gleditsiae Spina was added. The MTT was used to evaluate cell proliferation and viability and the medicinal properties of Gleditsiae Spina.

## 3. Results

### 3.1. Mass Spectrometry Results

**Figure 1:**
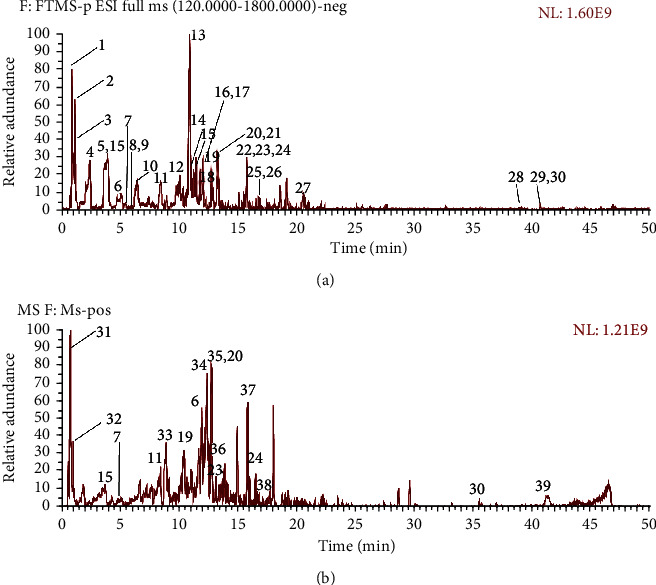
UHPLC-Q-Exactive Orbitrap MS identification results of the main chemical components in the extract of Gleditsiae Spina. (a) Total ion current graph in positive ion and negative ion mode. (b) Total ion current graph in negative ion and negative ion mode.

The extract of Gleditsiae Spina was analyzed according to the method in Section 2.1, and the total ion current diagram under the positive and negative ion mode was obtained (Figure 1). The height of the labels represents the relative content of ingredients. According to the retention time of each chemical component, high-resolution precise molecular weight, MSn multilevel fragment information obtained by LC-MS detection, combined with the extracted ion current map and standard product information, Mzcloud database, and related literature, the composition was confirmed, and a total of 39 were identified. The results were shown in Table 1, and the mass spectra of 39 identified compounds were shown in Supplementary material 1. Among them, the number identified in the positive ion mode is 30, the number identified in the negative ion mode is 18, and the 9 compounds are the compounds identified by the positive and negative ions.

### 3.2. Network Construction and Target Prediction

#### 3.2.1. Active Ingredients and Targets of Gleditsiae Spina

**Figure 2:**
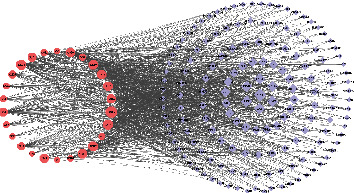
Active component-target network map of Gleditsiae Spina. The purple labels represent the targets of the action of active components in the figure; the red labels represent 26 Gleditsiae Spina active components.

Thirty-nine Gleditsiae Spina components were observed in Section 3.1, screened according to the pharmacokinetics and drug similarity in SwissADME platform, and supplemented according to previous literature reports. This resulted in 26 active components. The SDF structure of 26 selected components were obtained from PubChem, and the PharmMapper target prediction model was used to predict the above 26 targets of active ingredients. The 26 medicinal active ingredients are shown by in Table 2. Relevant target prediction technology was used to predict the active targets, eliminating duplicate targets, and a total of 610 predicted targets were obtained, as shown in Figure 2.

#### 3.2.2. Construction and Analysis of “Active Ingredient-Target” Network

Cytoscape 3.7.2 was used to draw and analyze the relationship network between the effective components of Gleditsiae Spina and its active targets, and a total of 635 nodes (including 609 targets and 26 active components) and 1090 relationships were obtained. The size represented the corresponding degree value. The larger the node area was, the larger the degree value, indicating that the more biological functions involved, the higher its biological importance (Figure 2).

#### 3.2.3. Ovarian Cancer-Related Target Searching

**Figure 3:**
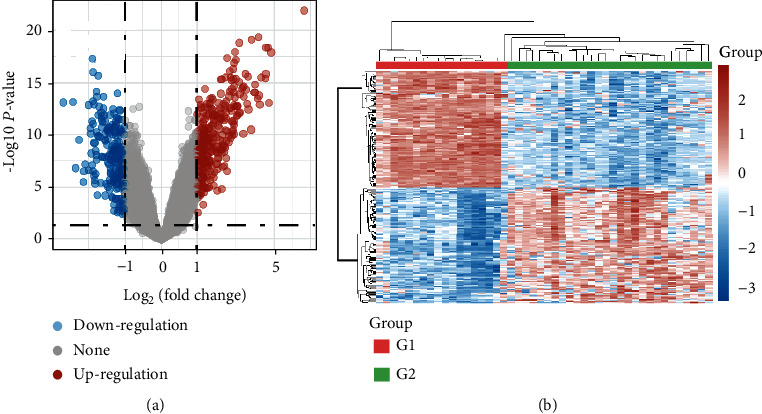
Volcano plot of differentially expressed genes. The abscissa represents the fold change in the gene expression, and the ordinate represents the statistical significance of the change in gene expression. Red dots represent genes with significantly increased expression, and blue dots represent genes with reduced expression (a). Heat map of differentially expressed genes. Heat map shows differentially expressed genes in normal ovarian tissue (G1) and HGSC (G2). Upregulated and downregulated genes are shown (b).

Ovarian cancer-related targets (1483) were identified from the Gene Expression Omnibus (GEO) database.  Figure 3 shows heat maps and volcano maps indicating the distribution of differentially expressed genes, which are represented by red dots on the map.

#### 3.2.4. Screening of Key Targets for Gleditsiae Spina Treatment in Ovarian Cancer

**Figure 4:**
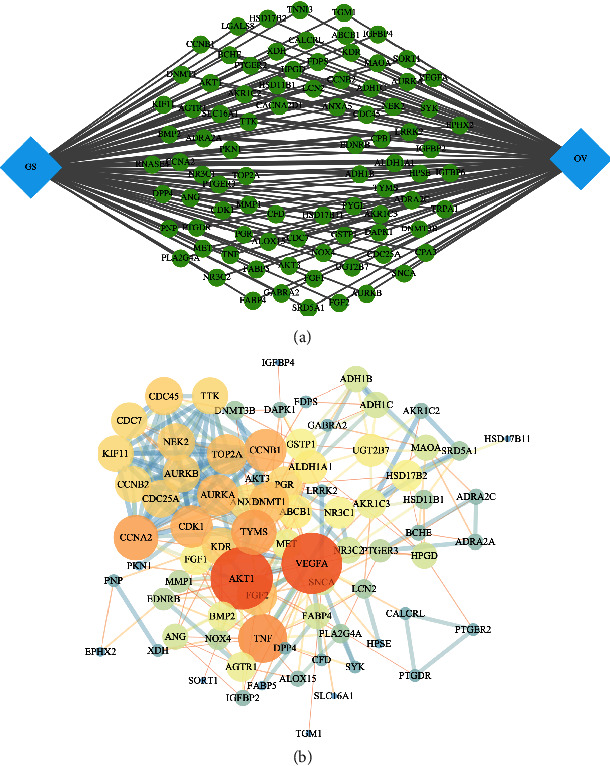
The key targets of the Gleditsiae Spina treating for ovarian cancer (a) and the PPI network (b).

To obtain richer node-node connection information in PPI networks, the efficiency of node information transmission was optimized, the targets that play an important role in the network were identified, and the network topology characteristic attributed values of the abovementioned intersection PPI network graph was calculated. Through two screenings, a total of 87 key targets were obtained. The targets were shown in Figure 4(a), and the PPI network of the key genes was shown in Figure 4(b).

#### 3.2.5. Pathway Enrichment Analysis and Visualization of Gleditsiae Spina Treatment for Ovarian Cancer

**Figure 5:**
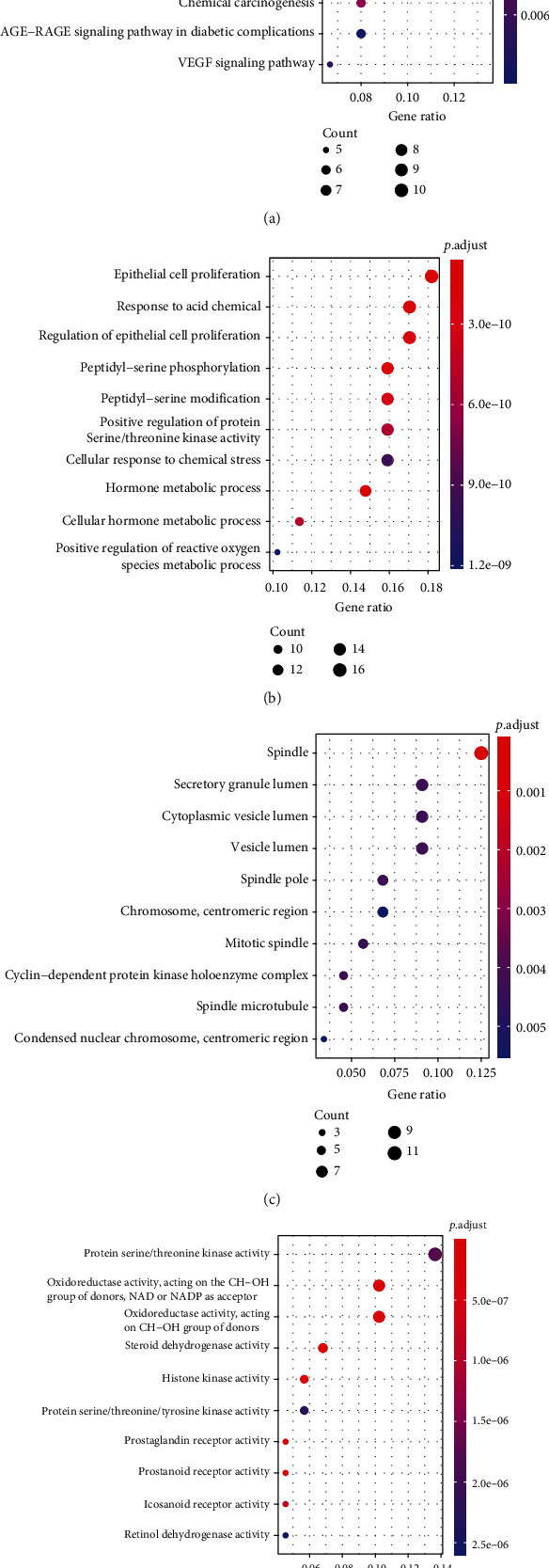
Bubble diagram of GO and KEGG enrichment of key targets for Gleditsiae Spina for treatment of HGSC. (a) KEGG analysis. (b) GO BP analysis. (c) GO CC analysis. (d) GO MF analysis. Pathways that had significant changes of log10 (*P*) < 0.05 were identified. Size of the spot represents number of genes, and color represents log10 (*P*) value.

Metascape platform was used to perform gene enrichment analysis on the above 87 key nodes, including GO-BP (biological process), GO-CC (cellular component), GO-MF (molecular function), and KEGG pathway. *R* (version 6.1) was used to draw KEGG pathway a bubble chart (shown in Figure 5). The bubble color changed from red to purple to indicate that the log *P* value is from small to large. The smaller the log10 (*P*) value, the stronger the significance, and the larger the bubble, the larger the gene count (count value) of the pathway.

#### 3.2.6. Molecular Docking and Molecular Dynamic Simulation Results

**Figure 6:**
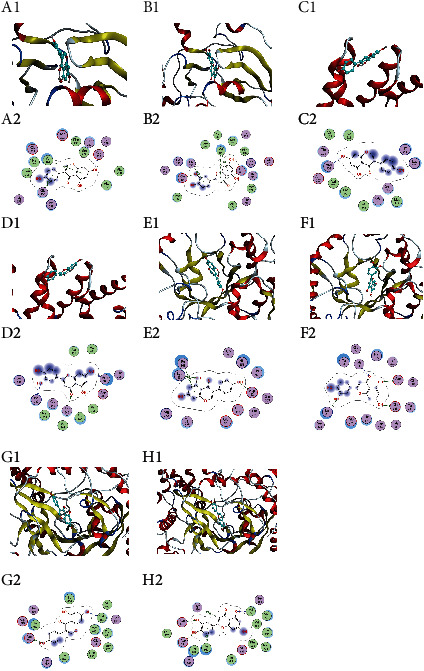
Molecular docking of compounds with core targets: (a) AKT1-genistein, (b) AKT1-luteolin, (c) CTNNB1-genistein, (d) CTNNB1-luteolin, (e) HPSE-genistein, (f) HPSE-luteolin, (g) PIK3CA-genistein, and (h) PIK3CA-luteolin.

The two core potential compounds luteolin and genistein were molecularly docked with the four core targets PIK3CA, CTNNB1, HPSE, and AKT1 to obtain the group receptor-ligand docking results. Among the nine groups, the highest docking score is for luteolin-HPSE (-8.97 kcal/mol), and the lowest docking score is for genistein-HPSE (-7.35 kcal/mol). This indicates that the selected potential core compounds have better binding activity with the target. The docking affinity value is shown in Table 3, and a diagram depicting eight docking modes is shown in Figure 6. It can be seen from the figure that each ligand is embedded in the active pocket of the target and that it interacts with multiple residues of the target through hydrophobic interactions and hydrogen bond formation.

**Figure 7:**
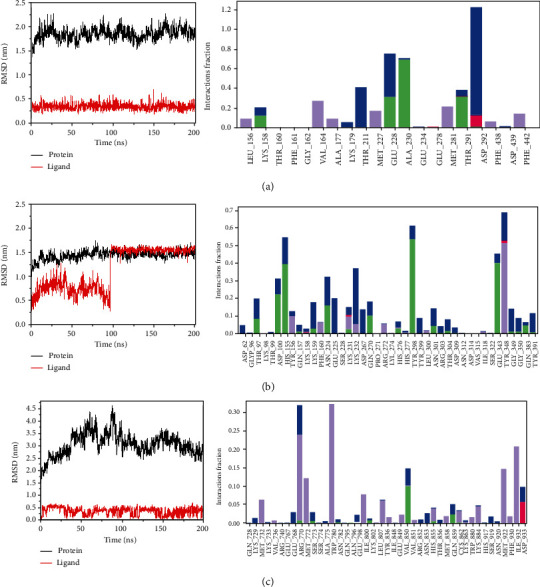
Molecular dynamic simulation of compounds with core targets: RMSD plot during molecular dynamic simulations of AKT1 with genistein (a), HPSE with luteolin (b), and PI3K with berberine (c).

Next, genistein, luteolin, and berberine as the core components in the network were selected to perform molecular dynamics simulation tests with AKT1, HPSE, and PI3K. The binding of genistein and luteolin to the ligand quickly stabilized and continued to work and after a short period of fluctuation, the binding of berberine to the ligand also forms a stable state. The results were shown in Figure 7.

### 3.3. Verification of Therapeutic Effects of Gleditsiae Spina on Ovarian Cancer

**Figure 8:**
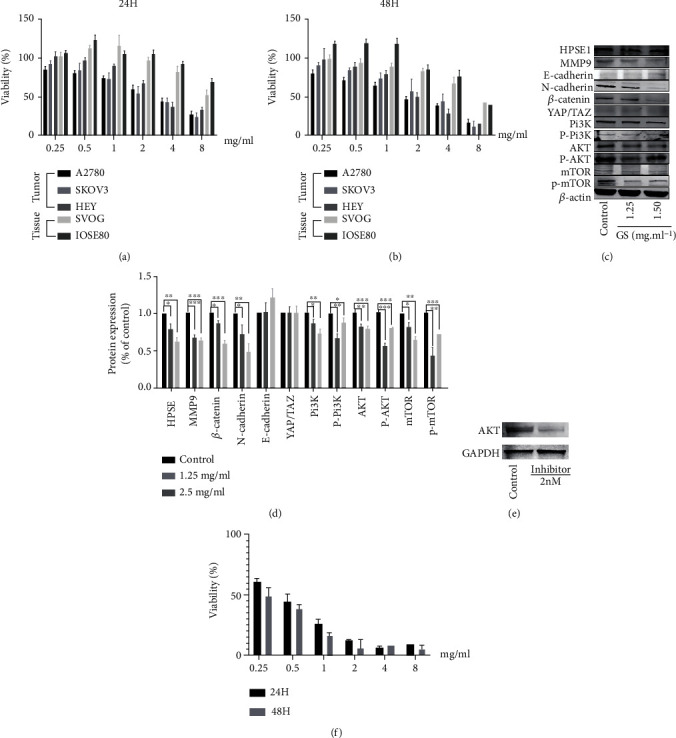
The cellular verification results. Gleditsiae Spina inhibited ovarian cancer cells proliferation in a dose-dependent way after incubation 24 h (a) and 48 h (b). (c) After exposure to the Gleditsiae Spina for 24 h, the total protein from each cell lysate was analyzed by Western blotting to measure the expression of proteins. (d) Data are presented as the mean ± SD and normalized to *β*-actin. (e) The AKT expression was decreased by AKT kinase inhibitor. (f) Dose-dependent inhibitory effect of Gleditsiae Spina on ovarian cancer cell proliferation under AKT kinase inhibitor of 2 nM (^∗^*P* < 0.05, ^∗∗^*P* < 0.01, ^∗∗∗^*P* < 0.001).

Next, we conducted experiments in vitro to verify the potential therapeutic targets of Gleditsiae Spina. The MTT assay was performed to evaluate the viability of A2780, SKOV3, and HEY cancer cells and normal cells IOSE80 and SVOG as comparison treated with increasing concentrations of Gleditsiae Spina solution (0.25-8 mg/mL) for 24 and 48 h. Gleditsiae Spina decreased ovarian cancer cells viability in a dose-dependent way, but not the normal ovarian cells, and the normal cells only showed a large amount of died when above 8 mg/mL. The results showed that the Gleditsiae Spina has a certain killing effect on tumor cells but not on tissue cells (shown in Figures 8(a) and 8(b)). To further explore the therapeutic mechanisms of Gleditsiae Spina in combination with the above-described KEGG analysis data, A2780 cells were stimulated with 1.25 and 2.5 mg/mL Gleditsiae Spina solution for 24 h, followed by protein extraction and analysis by western blotting, with heparinase as core (Figures 8(c) and 8(d)). Our results showed that the levels of heparinase 1, MMP9, *β*-catenin, and N-cadherin were significantly downregulated in ovarian cancer cells in response to Gleditsiae Spina treatment in a dose-dependent manner. However, the level of E-cadherin increased in the high-dose group; although, the increase was not statistically significant. Furthermore, treatment with Gleditsiae Spina inhibited the expression of proteins associated with the PI3K/AKT/mTOR pathway. However, the expression of these molecules increased at higher doses of Gleditsiae Spina compared to the low-dose group, though the higher doses of Gleditsiae Spina can also reduce the expression of PI3K/AKT kinase and their phosphorylated proteins This may be attributed to the interaction between the complex components of traditional Chinese medicine and the cells, which lead to cell death. At high Gleditsiae Spina concentrations, only a small number of cells survived for more than 48 h. Therefore, although the autocompensation mechanism of cells can be ruled out, the specific mechanism in vivo needs to be further explored. Next, we explored whether the antitumor effect of GS was AKT kinase-dependent. AKT kinase inhibitor was added to the cells at a concentration of 2 nM, and the expression of AKT kinase in ovarian cancer cells was shown in Figure 8(e). The antitumor effect of Gleditsiae Spina on A2780 cells after silencing AKT kinase expression is shown in Figure 8(f). After inhibiting the expression of AKT, the doubling time of cells increased from 26.3 hours to 30.2 hours, but the killing effect of Gleditsiae Spina on tumor cells also existed. The results showed that AKT is only one of the targets of Gleditsiae Spina, and the antitumor effect of Gleditsiae Spina partially depends on AKT kinase.

## 4. Discussion

Traditional Chinese medicine has a long history and has nurtured the Chinese nation for millennia. It protects human health through dialectical theory. Traditional Chinese medicine works against diseases through a single or compound prescription by multiple ingredients and targets. The cause of ovarian cancer is considered to be the accumulation of pathological products in TCM theory, and Gleditsiae Spina has the potential to clear these toxic metabolites. It is a summary of experience obtained in clinical practice of TCM and has obvious clinical effects based on clinical experience. The concept of network pharmacology and the theory of TCM have similar aspects, which can explore the unknown mechanism of action of Chinese herbal medicine from the perspective of overall composition and function [25]. Our experimental results also showed that Gleditsiae Spina has a selective therapeutic effect on ovarian cancer.

Based on the mass spectrometry results, along with previously reported data, we inferred that luteolin, genistein, D-(+)-tryptophan, ursolic acid, and berberine in the Gleditsiae Spina play a core role in the treatment of ovarian cancer. It was previously reported that D-(+)-tryptophan exhibits anticancer activity, which can stimulate mTORC1 and enhance the activity of T-cells within the tumor microenvironment [26]. Luteolin is a flavonoid compound that inhibits tumor cell proliferation, blocks cell cycle, and reverses tumor epithelial-mesenchymal transition [27]. Luteolin downregulates the expression of aromatase and consequently inhibits estrogen synthesis in ovarian cancer [28]. Genistein can modulate the cell cycle and regulate the ERK1/2, NF-*κ*B, Wnt, *β*-catenin, and PI3K/Akt signaling pathways to exert its anticancer effects. Moreover, it can synergize with paclitaxel and other ovarian cancer drugs [29]. Ursolic acid can downregulate the expression of YAP1 of the hippo pathway in tumor treatment [30]. Studies have shown that berberine can induce apoptosis in various tumor cell lines. By inhibiting the transcriptional activity of *β*-catenin, berberine modulates the Wnt signaling pathway [31] and increases the expression of caspase-3 and -8; thus, it promotes apoptosis of ovarian cancer cells, when used in combination with cisplatin [32].

The KEGG results showed that Gleditsiae Spina affects ovarian cancer development via multiple pathways and thus plays a therapeutic role. The expression levels of heparinase 1, MMP9, *β*-catenin, N-cadherin, and PI3K/AKT/mTOR, as well as their phosphorylation levels, which are involved in tumor progression, were reduced after treatment with Gleditsiae Spina.

Proteoglycans are widely distributed on the cell surface and in the cytoplasmic matrix; moreover, they play an important role in tumor development. Their glycosaminoglycan chains are modulated by heparinase, the only endoglycosidase in mammals; hence, heparinase plays an important role in regulating the function of proteoglycans [33]. Several studies have found that the expression of heparinase is positively correlated with malignancy, with heparinase overexpressing tumors being associated with a worse prognosis compared with tumors in which heparinase is under expressed [34].The extracellular matrix is mainly composed of keratan sulfate proteoglycans, chondroitin sulfate proteoglycans, and dermatan sulfate proteoglycans [35]; therefore, heparinase can accelerate the remodeling of tumor extracellular matrix and basement membrane [36] and contribute to tumor. Moreover, the cleaved heparan sulfate proteoglycans can bind to growth factors and increase the expression of VEGF by promoting p38 phosphorylation and Src kinase activity [37], thereby promoting angiogenesis and accelerating tumor metastasis and invasion [38]. Our in vitro experiments showed that Gleditsiae Spina can inhibit the tumor growth and downregulate heparinase expression in tumor cells. By reducing the expression of heparinase, Gleditsiae Spina can regulate signaling cascades within the tumors [39].

MMP9 plays an important role in tumor extracellular matrix remodeling and communication between tumor cells [40]. MMP9 is considered a potential biomarker for tumors, including ovarian cancer [41]. Our western blotting results showed that Gleditsiae Spina significantly inhibits the expression of MMP9 to reduce tumor cell activity. E-cadherin is a calcium ion-dependent transmembrane protein closely related to cell adhesion. Adjacent cells interact via an extracellular domain of E-cadherin, which is connected to *β*-catenin and the cell cytoskeleton. Thus, the E-cadherin expression is negatively correlated with the degree of tumor invasion [42]. Numerous studies have shown that the conversion of E-cadherin to N-cadherin often indicates the completion of the epithelial–mesenchymal transition process. Herein, although the expression of E-cadherin was low and changes in its expression could not be measured, the expression of N-cadherin was inhibited after treatment with Gleditsiae Spina, indicating that Gleditsiae Spina can reverse the expression of the two proteins, thereby inhibiting tumor development. *β*-Catenin is a cytoplasmic protein, and its nuclear expression plays a role in activating transcription factors. The abnormal expression of *β*-catenin is related to the hippo and HIF1 signaling pathways, and it can also activate the Wnt signaling pathway [43]. Moreover, its abnormal expression is closely related to colon cancer [44] and cervical cancer. Our western blotting results showed that Gleditsiae Spina inhibited the expression of *β*-catenin protein, which were consistent with the prediction results of the network pharmacology.

The PI3K/AKT/mTOR signaling pathway plays key role in cancer, as is related to cell proliferation, angiogenesis, chemotherapy resistance, and several other pathological conditions. Its downstream molecule mTOR can accelerate the formation of tumor stem cells [45], leading to tumor progression and relapse. The overexpression of PI3K can lead to RAS mutations and loss of PTEN and is associated with HIF1, hippo, and MAPK signaling pathways. Moreover, it activates the epidermal growth factor receptor, stimulates the expression of the vascular endothelial growth factor, and accelerates angiogenesis [46]. Approximately 70% of ovarian cancer patients present with overexpressed PI3K/AKT/mTOR cascade [47]; thus, using inhibitors to target this pathway is an important strategy to treat ovarian cancer. In our study, Gleditsiae Spina significantly inhibited the expression of PI3K and AKT; low-dose treatment with Gleditsiae Spina inhibited the phosphorylation of AKT and mTOR, whereas the remarkably increased phosphorylation in the high-dose-treated group suggested its own S6K1-IRS1 negative feedback. Additional experiments are warranted to elucidate the underlying mechanism of the regulatory effect exerted by Gleditsiae Spina. According to the WB results, when the cancer cells were exposed at the concentration of 2.5 mg/mL of Gleditsiae Spina, the levels of AKT and P-AKT kinase expressed in cells are downregulated, and the phosphorylated protein is not significantly decreased. The results indicated that Gleditsiae Spina did not mainly affect the phosphorylation of AKT pathway proteins, but its complex biological effects reduced the Pi3k/AKT and its phosphorylation kinase in cells. Later, we want to know whether the therapeutic effect of Gleditsiae Spina was dependent on the AKT pathway. We inhibited the expression of AKT in cells. In this case, Gleditsiae Spina still had an inhibitory effect, which also proves our prediction that PI3K/AKT signaling pathway is only one of the effects of Gleditsiae Spina. Traditional Chinese medicine has a complex mechanism in the treatment of ovarian cancer. The characteristics of multitarget and multipathway interaction need to be explored.

The molecular docking experiments can predict the binding ability of the ligand and the target at the molecular level. Using this approach, we determined that several components of Gleditsiae Spina have high binding ability to the ovarian cancer targets. Gleditsiae Spina can interfere with the activities of heparinase 1, *β*-catenin, PI3K, and AKT at the molecular level, thereby proving that Gleditsiae Spina can exert significant therapeutic effects on ovarian cancer. Molecular dynamic simulations, which can monitor time-resolved motions of molecules [48], further showed that the ingredients of Gleditsiae Spina stably interact with the disease-specific molecules in ovarian cancer, which indirectly provides a basis for verifying its therapeutic efficacy.

## 5. Conclusion

In this study, we firstly clarified the main components of Gleditsiae Spina and then predicted potential targets through network pharmacology and verified through molecular docking, molecular dynamic simulation, and experimental verification in vitro. We found that Gleditsiae Spina can regulate PI3K/AKT pathway and the composition of cytoplasmic matrix and proteoglycans in cancer. The regulatory mechanism of Gleditsiae Spina on ovarian cancer was partially revealed in this study, laying a foundation for future scientific research on TCM.

## Figures and Tables

**Table 1:** UPLC-MS/MS analysis results of the main chemical components of Gleditsiae Spina.

Peak number	*t*R/min	Molecular formula	Accurate	Calculated	Error (ppm)	Identified compounds
1.	0.824	C_6_H_12_O_6_	180.06339	180.0629	−2.48	D-(+)-Glucose
2.	1.018	C_4_H_6_O_5_	134.02152	134.0211	−2.92	DL-malic acid
3.	1.229	C_6_H_6_O_3_	126.03169	126.0312	−4	Pyrogallol
4.	2.201	C_9_H_17_NO_5_	219.11067	219.1102	−2.14	Pantothenic acid
5.	3.265	C_8_H_8_O_3_	152.04734	152.047	−2.3	2-Anisic acid
6.	4.062	C_7_H_6_O_3_	138.03169	138.0313	−3.07	Salicylic acid
7.	5.626	C_15_H_14_O_6_	290.07904	290.0787	−1.33	Catechin
8.	5.72	C_6_H_10_O_4_	146.05791	146.0576	−2.4	Adipic acid
9.	5.763	C_8_H_8_O_4_	168.04226	168.0419	−2.36	Vanillic acid
10.	6.578	C_7_H_6_O_2_	122.03678	122.0365	−2.45	Benzoic acid
11.	8.569	C_8_H_8_O_3_	152.04734	152.047	−2.15	Vanillin
12.	9.315	C_10_H_10_O_4_	194.05791	194.0575	−2.29	Ferulic acid
13.	10.946	C_15_H_12_O_7_	304.0583	304.0576	−2.21	Dihydrorobinetin
14.	10.953	C_6_H_6_O_3_	126.03169	126.0309	−5.95	Phloroglucinol
15.	4.049	C_11_H_12_N_2_O_2_	204.08988	204.0894	−2.25	D-(+)-Tryptophan
16.	11.22	C_21_H_20_O_10_	432.10565	432.1049	−1.85	Isovitexin
17.	11.275	C_21_H_20_O_12_	464.09548	464.0949	−1.25	Quercetin-3-O-glucoside
18.	12.071	C_9_H_8_O_2_	148.05243	148.0521	−2.07	trans-Cinnamic acid
19.	12.774	C_21_H_20_O_11_	448.10056	448.0998	−1.67	Isoorientin
20.	12.895	C_15_H_12_O_6_	288.06339	288.0629	−1.64	Fustin
21.	13.052	C_9_H_10_O_2_	150.06808	150.0678	−2.18	Hydrocinnamic acid
22.	15.655	C_8_H_11_NO	137.08406	137.0837	−2.6	Tyramine
23.	15.736	C_15_H_10_O_7_	302.04265	302.042	−2.3	Robinetin
24.	15.751	C_15_H_10_O_6_	286.04774	286.0472	−2.06	Luteolin
25	17.106	C_9_H_16_O_4_	188.10486	188.1044	−2.64	Azelaic acid
26.	17.631	C_15_H_10_O_5_	270.05282	270.0525	−1.36	Genistein
27.	21.755	C_16_H_30_O_4_	286.21441	286.2139	−1.63	Hexadecanedioic acid
28.	38.385	C_30_H_46_O_4_	470.33961	470.3389	−1.43	18-*β*-Glycyrrhetinic acid
29.	40.138	C_30_H_48_O_3_	456.36035	456.3597	−1.36	Ursolic acid
30.	41.117	C_30_H_48_O_3_	456.36035	456.36	−0.83	Oleanolic acid
31.	0.753	C_6_H_14_N_4_O_2_	174.11168	174.11157	−0.58	L-(+)-Arginine
32.	1.118	C_9_H_11_NO_3_	181.07389	181.07368	−1.17	L-tyrosine
33.	9.58	C_10_H_10_O_2_	162.06808	162.0679	−1.1	Methyl cinnamate
34.	11.63	C_9_H_7_NO	145.05276	145.05256	−1.41	8-Hydroxyquinoline
35.	11.787	C_21_H_20_O_10_	432.10565	432.10494	−1.64	Vitexin
36.	13.668	C_10_H_16_O	152.12012	152.12	−0.75	Citral
37.	16.6	C_20_H_17_NO_4_	335.11576	335.11495	−2.42	Berberine
38.	17.966	C_16_H_12_O_7_	316.0583	316.05771	−1.89	Rhamnetin
39.	43.089	C_29_H_48_O_2_	428.36543	428.36437	−2.48	Stigmastane-3,6-dione

**Table 2:** Candidate active components in Gleditsiae Spina.

ID	Mol name
GS1	Adipic acid
GS2	Azelaic acid
GS3	Berberine
GS4	Catechin
GS5	Citral
GS6	D-(+)-Glucose
GS7	D-(+)-Tryptophan
GS8	Ferulic acid
GS9	Fustin
GS10	Genistein
GS11	Hexadecanedioic acid
GS12	Hydrocinnamic acid
GS13	Luteolin
GS14	Methyl cinnamate
GS15	Oleanolic acid
GS16	Pantothenic acid
GS17	Phloroglucinol
GS18	Rhamnetin
GS19	Robinetin
GS20	Salicylic acid
GS21	Stigmastane-3,6-dione
GS22	trans-Cinnamic acid
GS23	Tyramine
GS24	Ursolic acid
GS25	Vanillic acid
GS26	Vanillin

**Table 3:** The docking affinity value of compounds with core targets.

	Luteolin	Genistein
PIK3CA	-8.49	-8.59
CTNNB1	-8.47	-7.56
HSPE	-8.97	-7.35
AKT1	-8.22	-8.79

## Data Availability

The data that support the findings of this study are available from the corresponding authors upon reasonable request.

## Abbreviation

ERK: Extracellular signal-regulated kinases
HGSC: High-grade serous ovarian cancer
HPLC: High performance liquid chromatography
MCE: MedChemExpress
MDSCs: Myeloid-derived suppressor cells
MMP9: Matrix metalloproteinase-9
MS: Mass spectrometry
MTT: 3-(4,5-Dimethylthiazol-2-yl)-2,5-diphenyltetrazoliumbromide
RMSD: Root mean square deviation
RMSF: Root mean square fluctuation
ROS: Reactive oxygen species
TCM: Traditional Chinese medicine
TNF-*α*: Tumor necrosis factor-alpha
WB: Western blot.

## Supplementary Materials

Supplementary Materials Supplementary material 1: the mass spectra of 39 identified compounds was shown in supplementary material 1. Through the mass spectrum of each component, the structure of the compound could be speculated, and the type of compounds was indirectly determined.
