# Supplementary material for: Molecular Mechanism of Gleditsiae Spina for the Treatment of High-Grade Serous Ovarian Cancer Based on Network Pharmacology and Pharmacological Experiments
Source: Biomed Res Int. 2022 Mar 8;2022:5988310. doi: 10.1155/2022/5988310 (PMC8923798; doi:10.1155/2022/5988310)

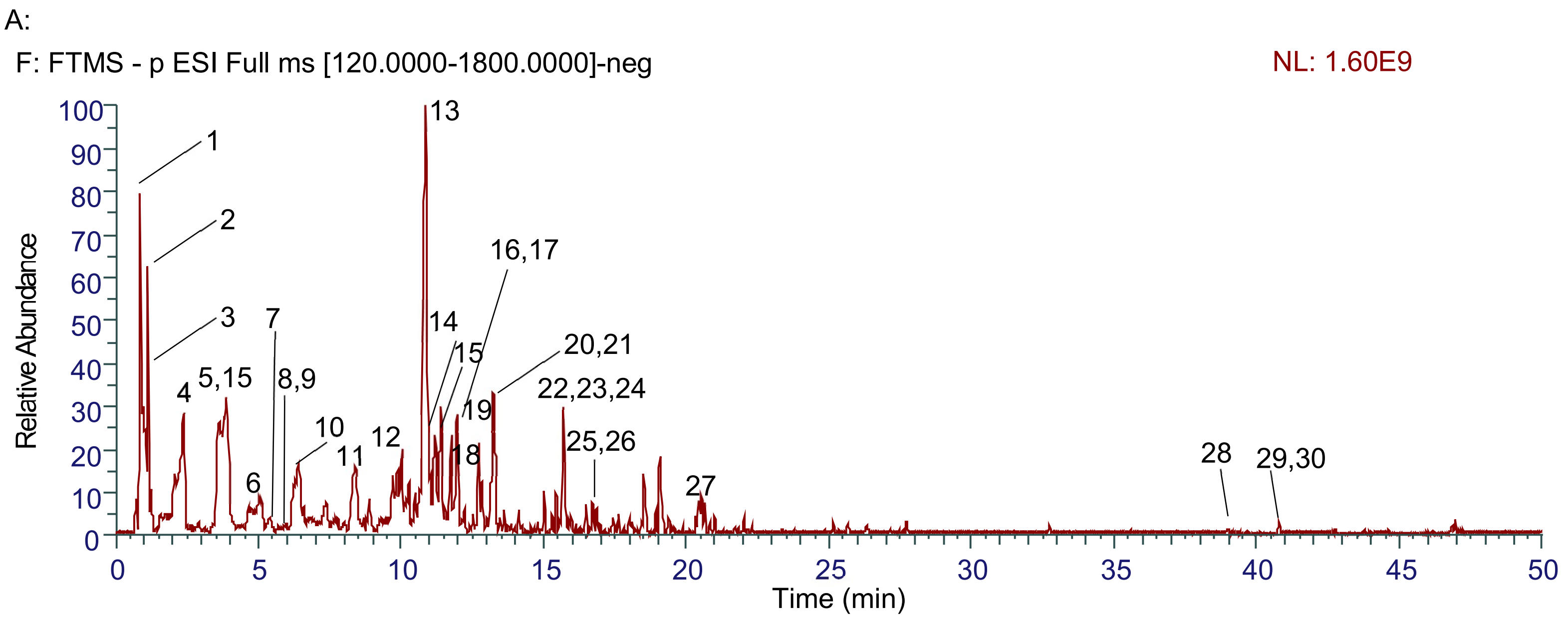

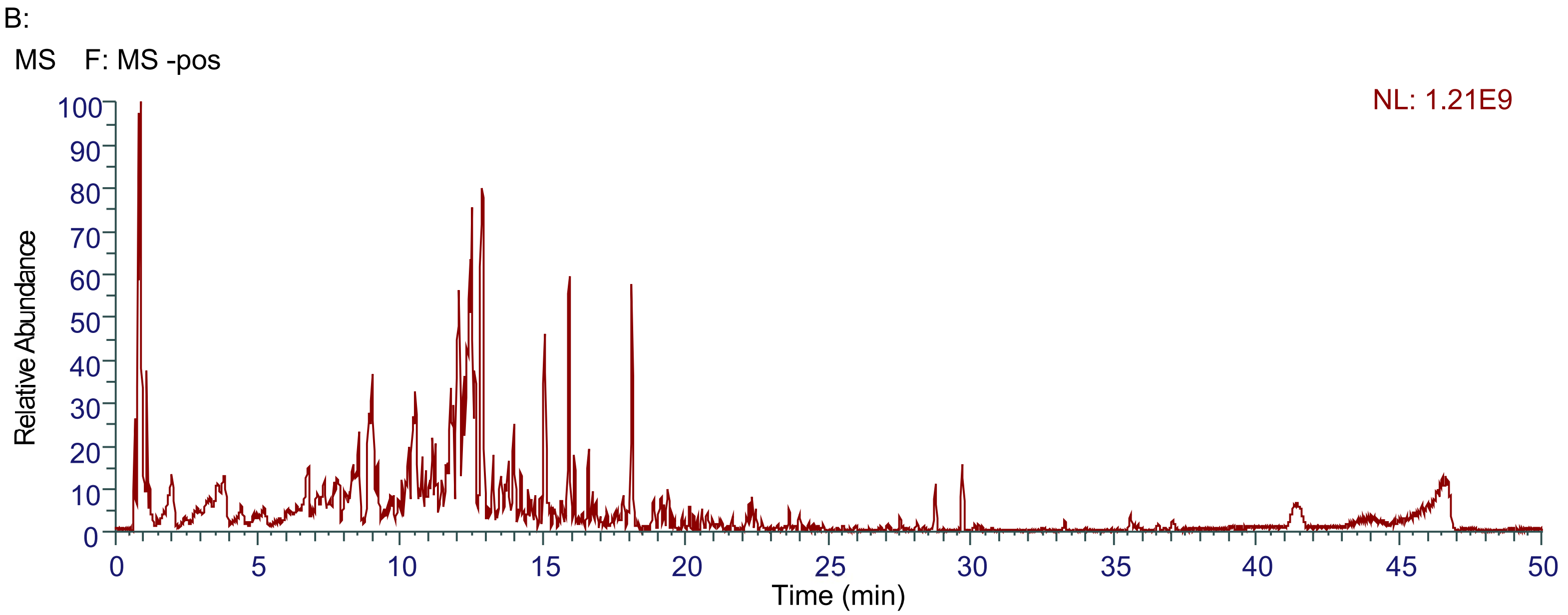

UHPLC-Q-Exactive Orbitrap MS identification results of the main chemical components in the extract of Gleditsiae Spina. A total ion current graph in positive ion and negative ion mode; B total ion current graph in negative ion and negative ion mode.

1. D-(+)-Glucose

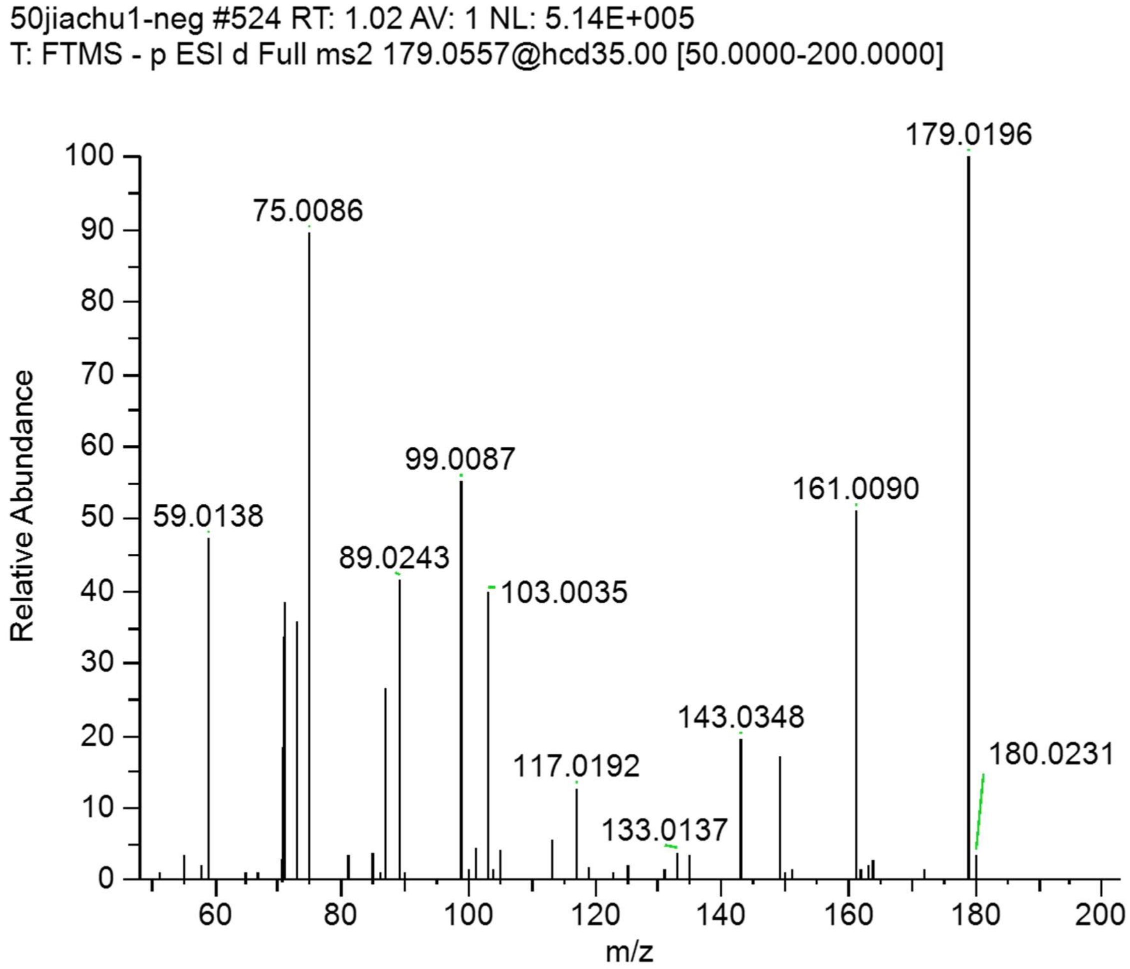
50jiachu1-neg #524 RT: 1.02 AV: 1 NL: 5.14E+005
T: FTMS - p ESI d Full ms2 179.0557@hcd35.00 [50.0000-200.0000]
179.0196
75.0086
99.0087
161.0090
59.0138
89.0243
103.0035
143.0348
117.0192
180.0231
133.0137
Relative Abundance
m/z

2. DL-Malic acid

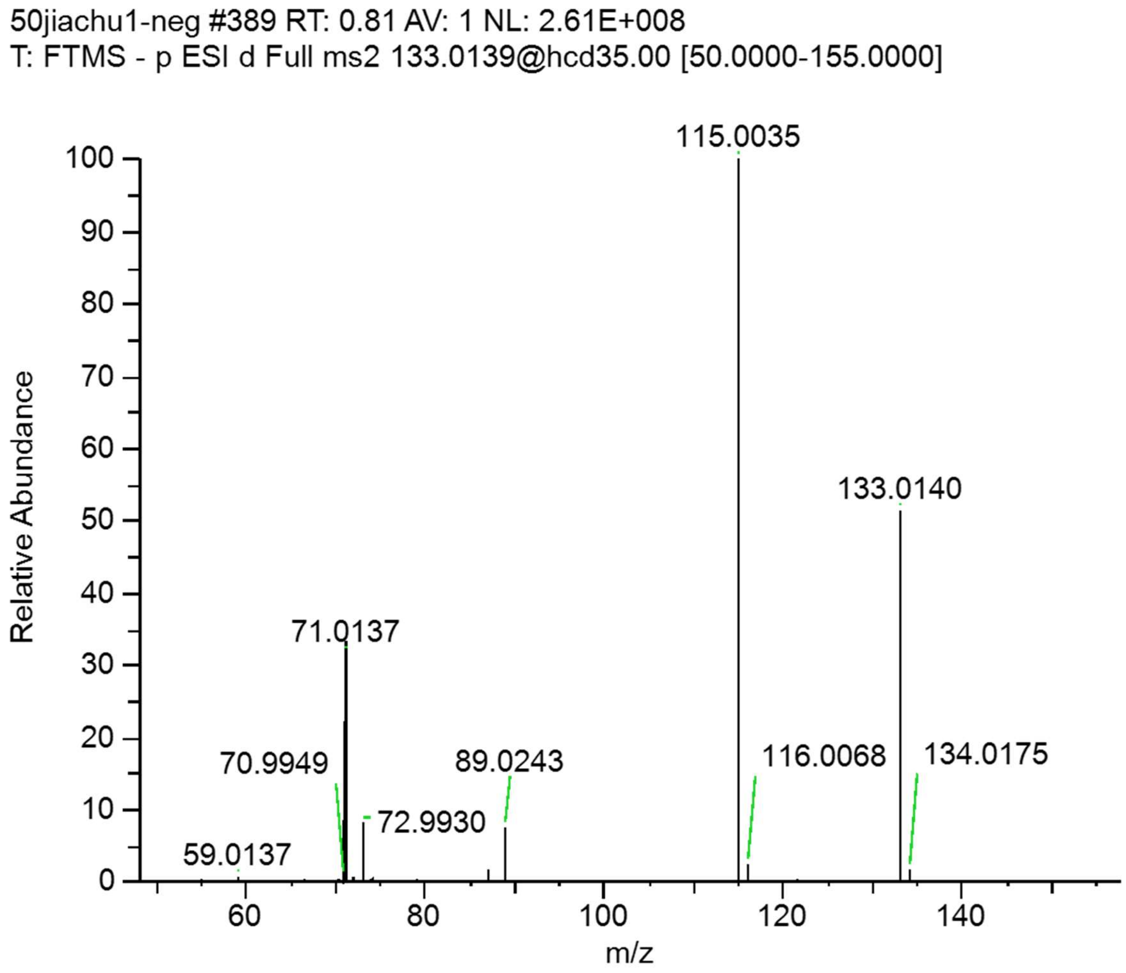

3. Pyrogallol

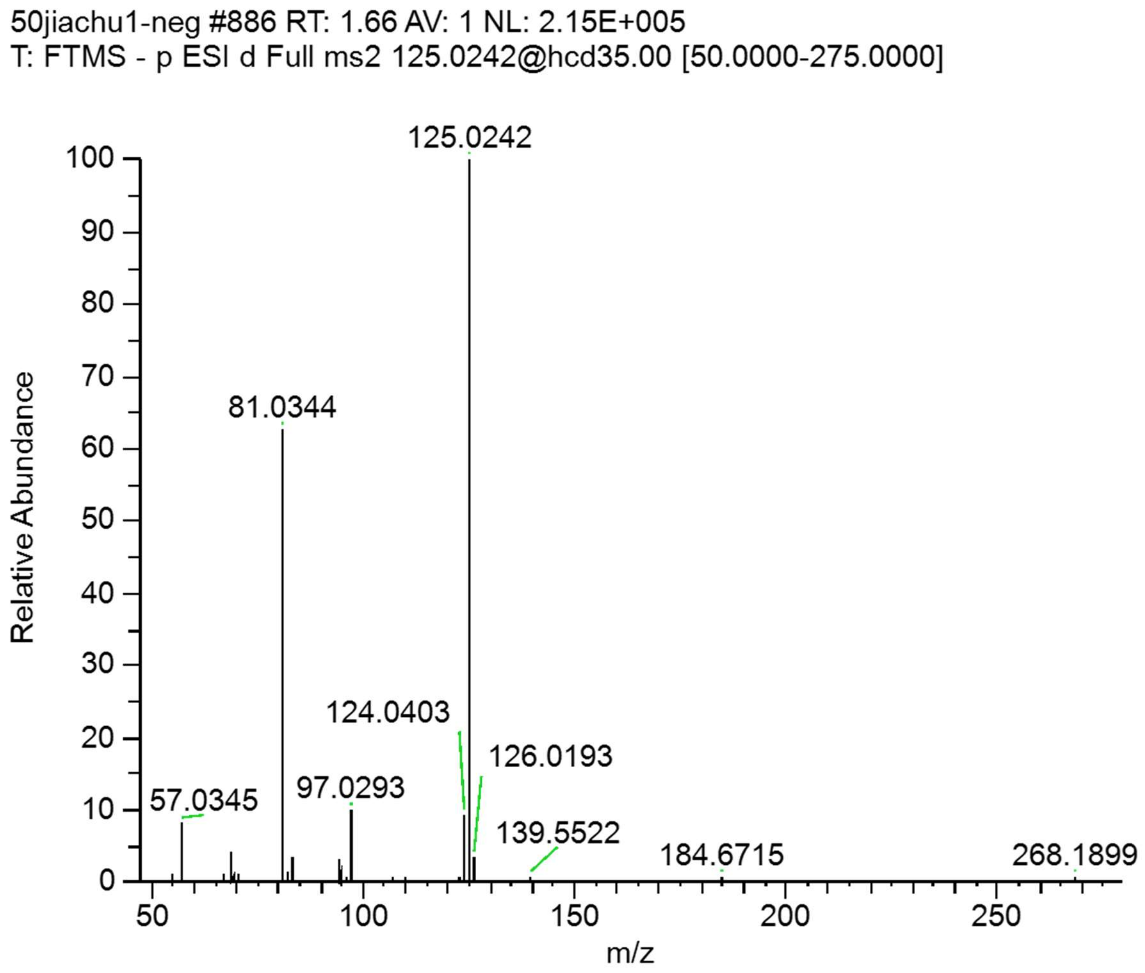

4. Pantothenic acid

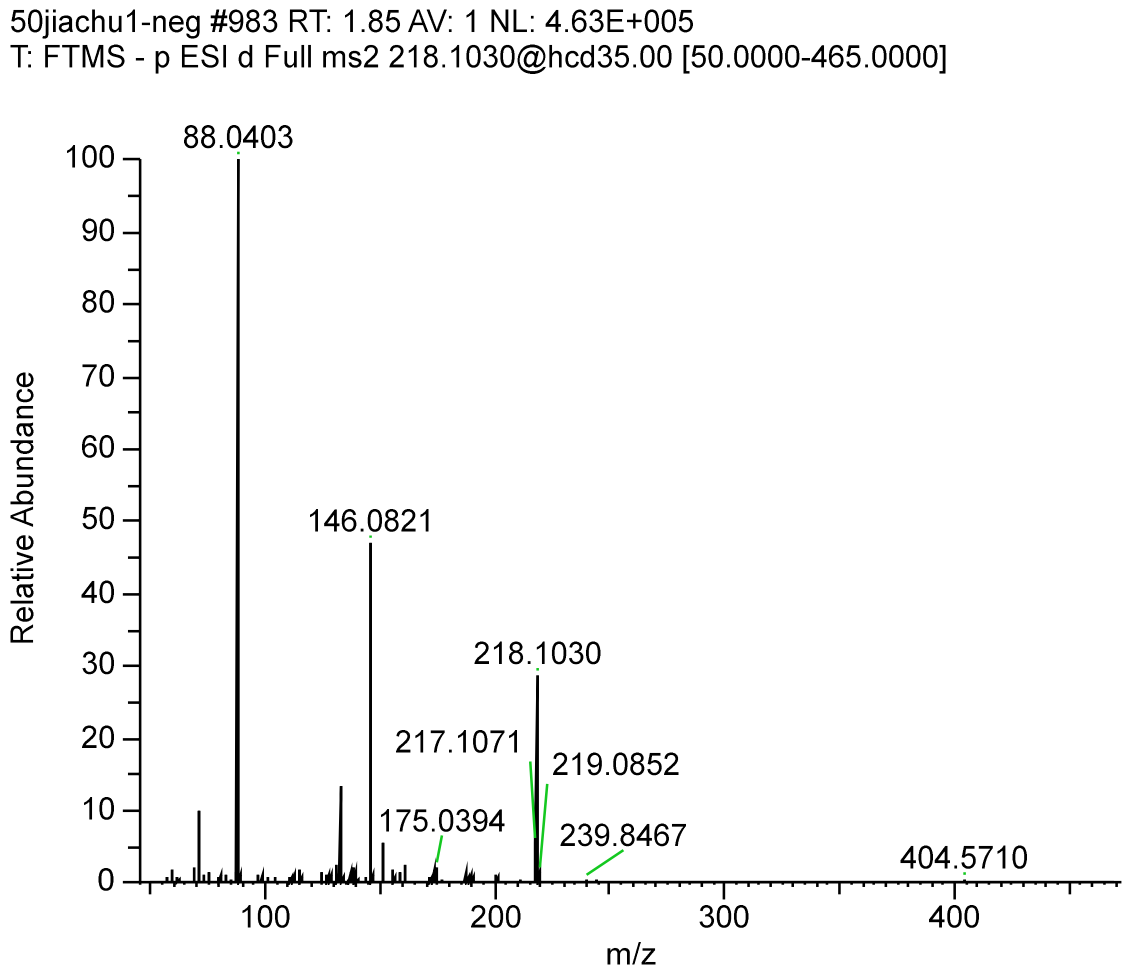

5. 2-Anisic acid

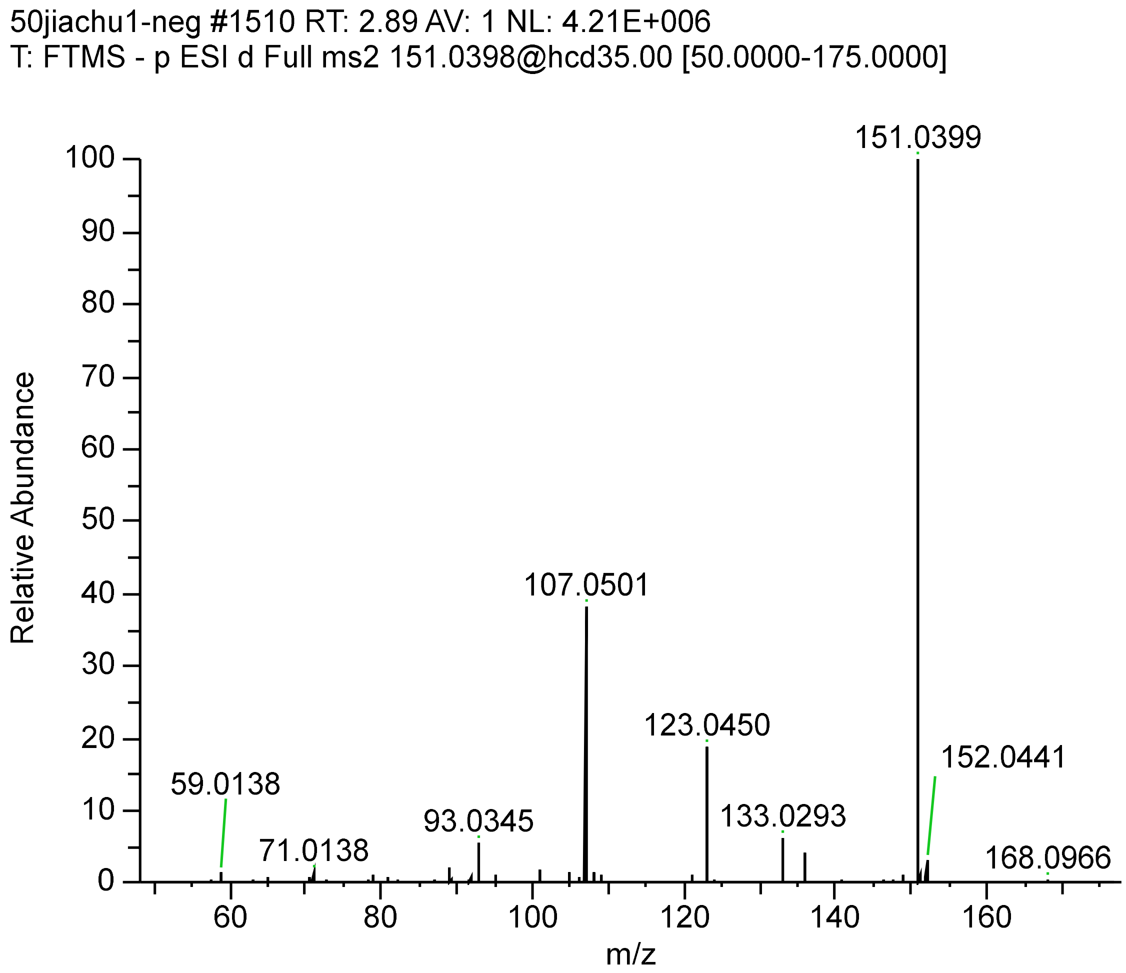

6. Salicylic acid

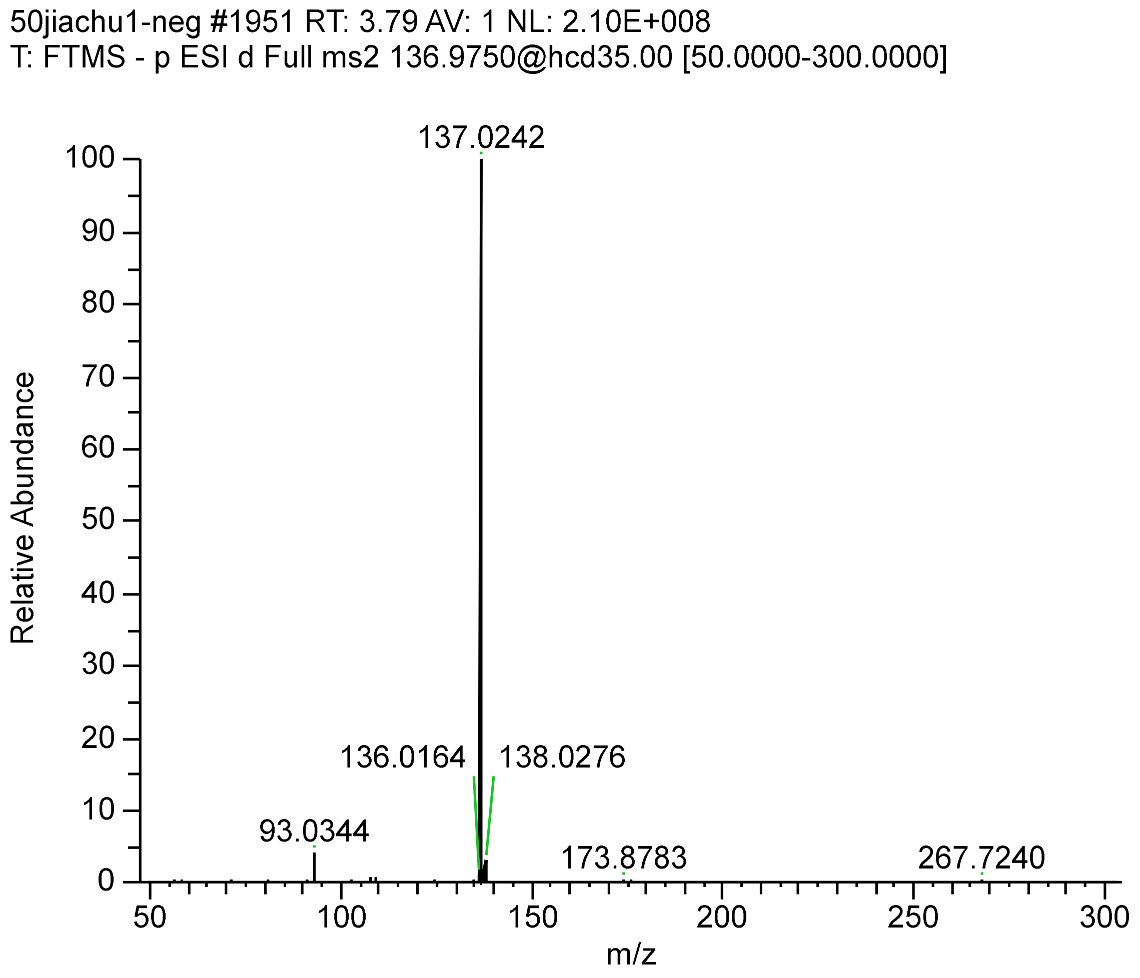

7. Catechin

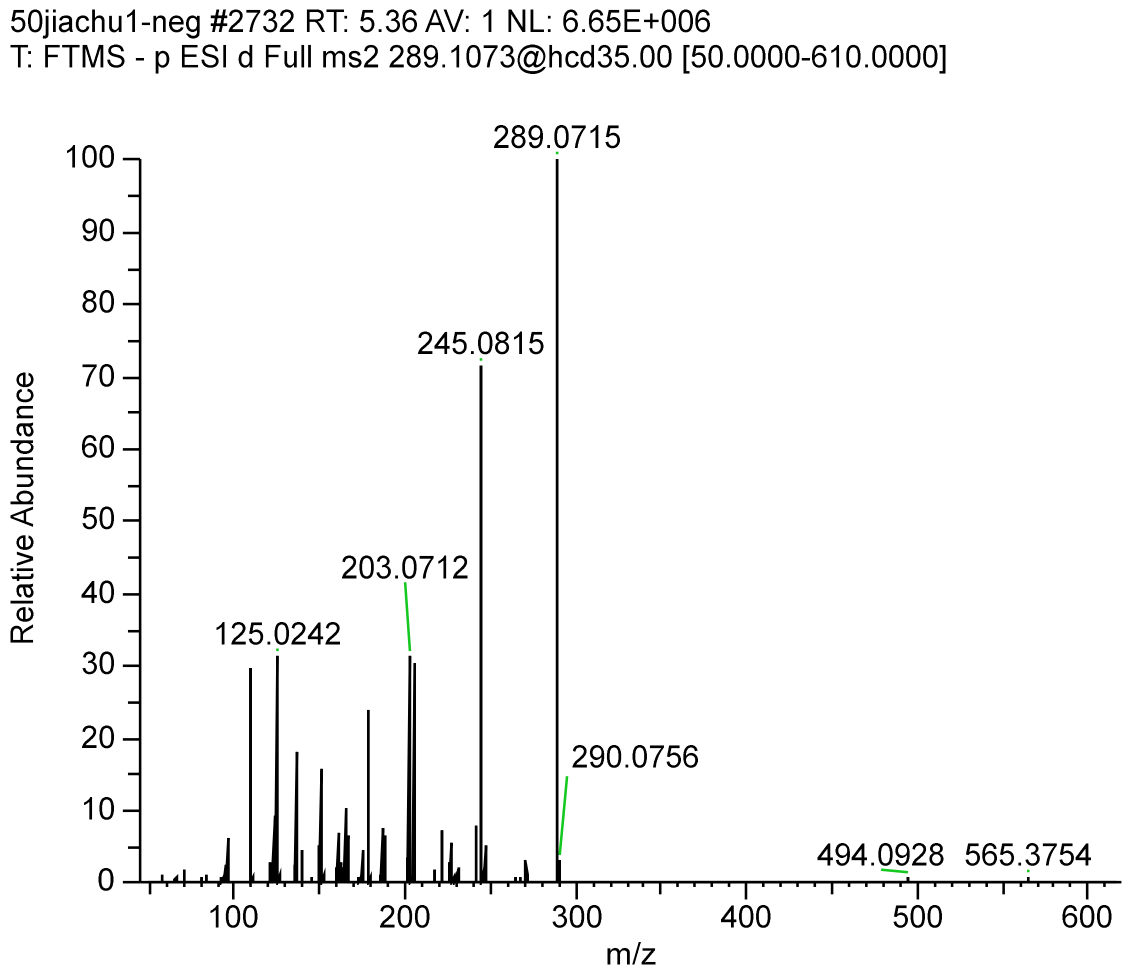
50jiachu1-neg #2732 RT: 5.36 AV: 1 NL: 6.65E+006
T: FTMS - p ESI d Full ms2 289.1073@hcd35.00 [50.0000-610.0000]
289.0715
245.0815
203.0712
125.0242
290.0756
494.0928
565.3754
Relative Abundance
m/z
0
10
20
30
40
50
60
70
80
90
100
200
300
400
500
600

8. Adipic acid

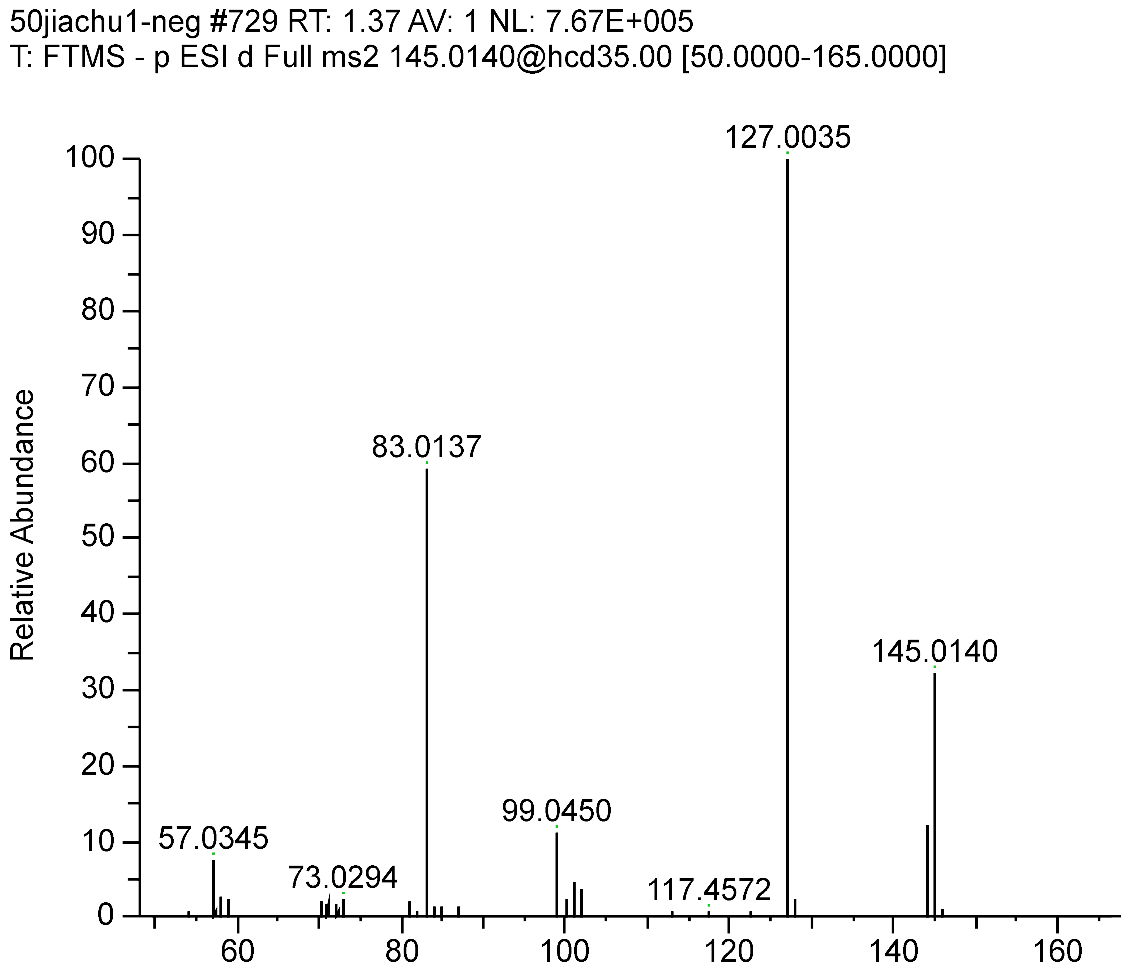

9. Vanillic acid

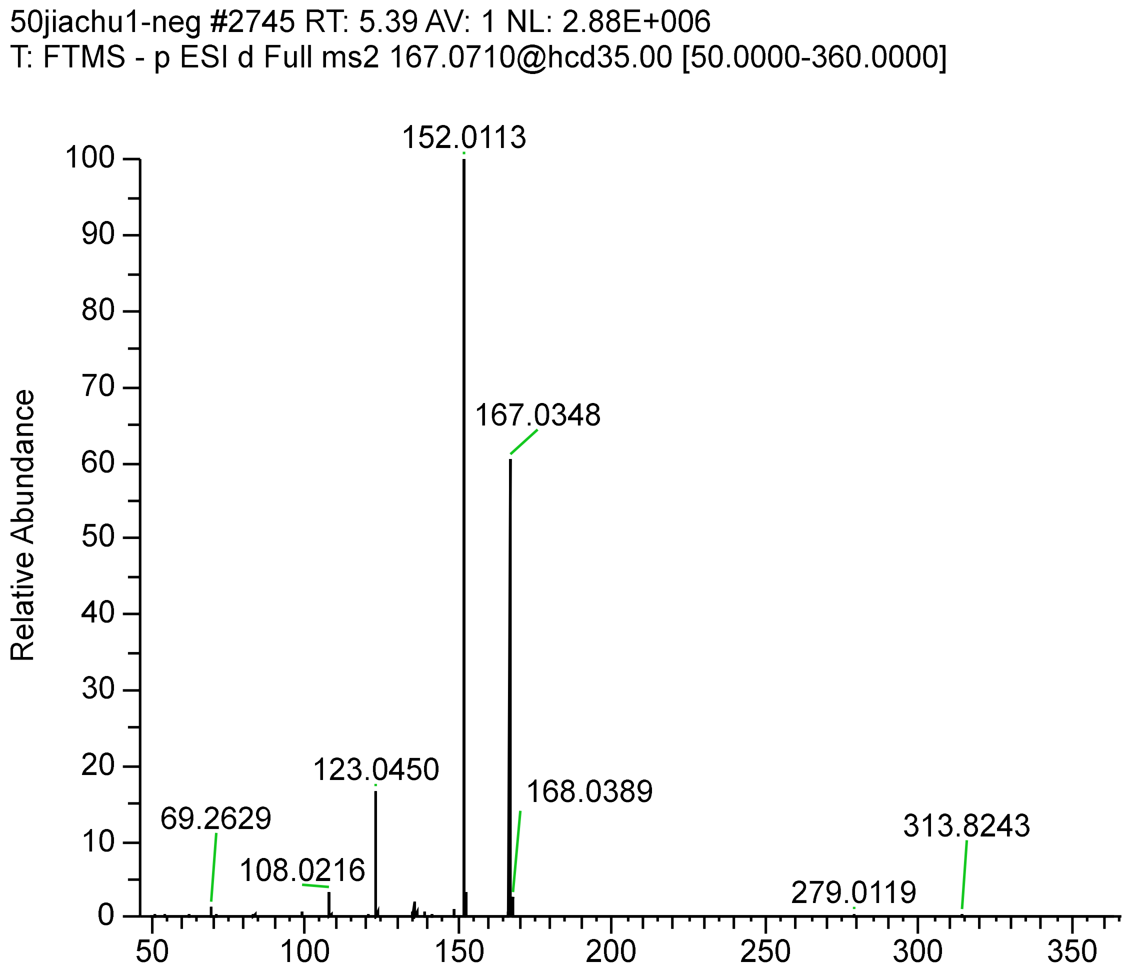

10. Benzoic acid

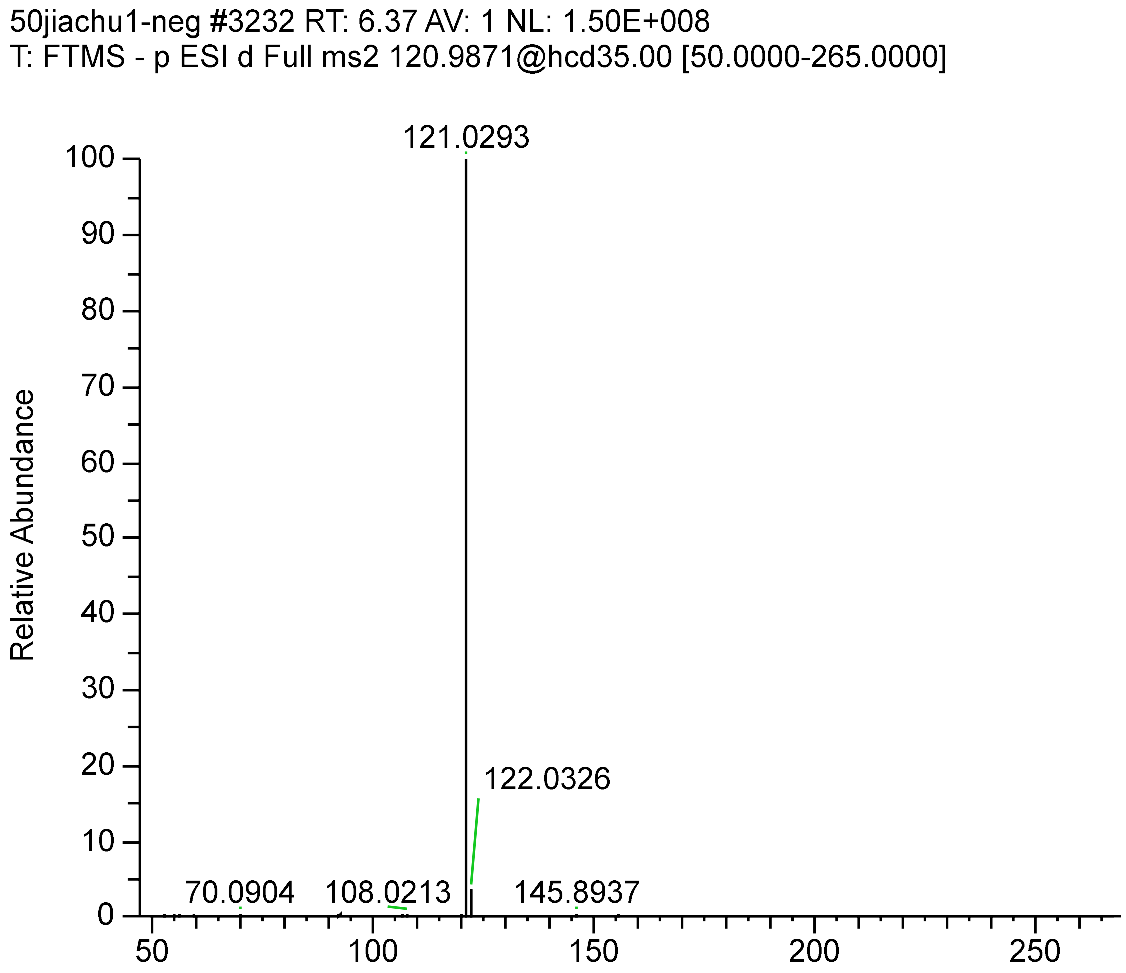

11. Vanillin

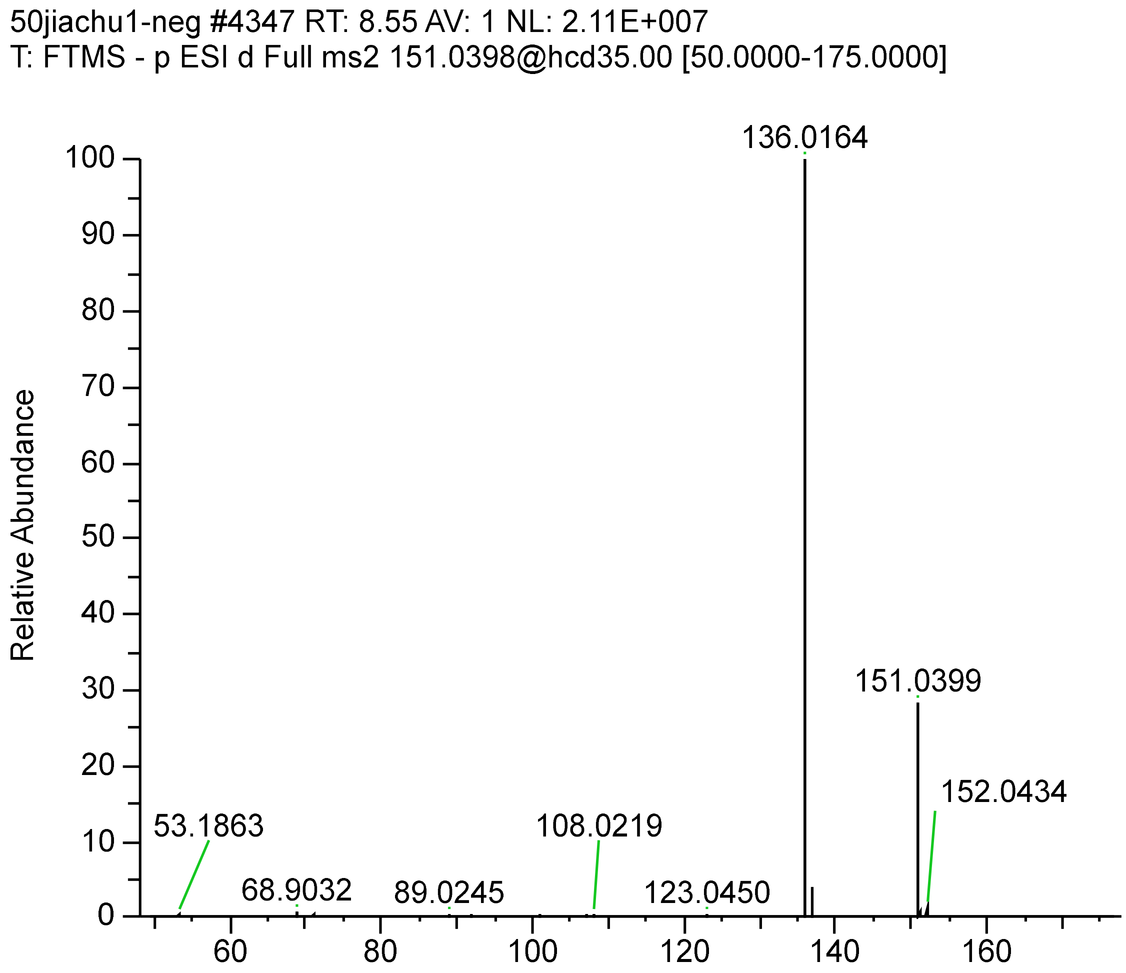

50jiachu1-neg #4347 RT: 8.55 AV: 1 NL: 2.11E+007
T: FTMS - p ESI d Full ms2 151.0398@hcd35.00 [50.0000-175.0000]
Relative Abundance
136.0164
151.0399
152.0434
53.1863
68.9032
89.0245
108.0219
123.0450
0
10
20
30
40
50
60
70
80
90
100
60
80
100
120
140
160

12. Ferulic acid

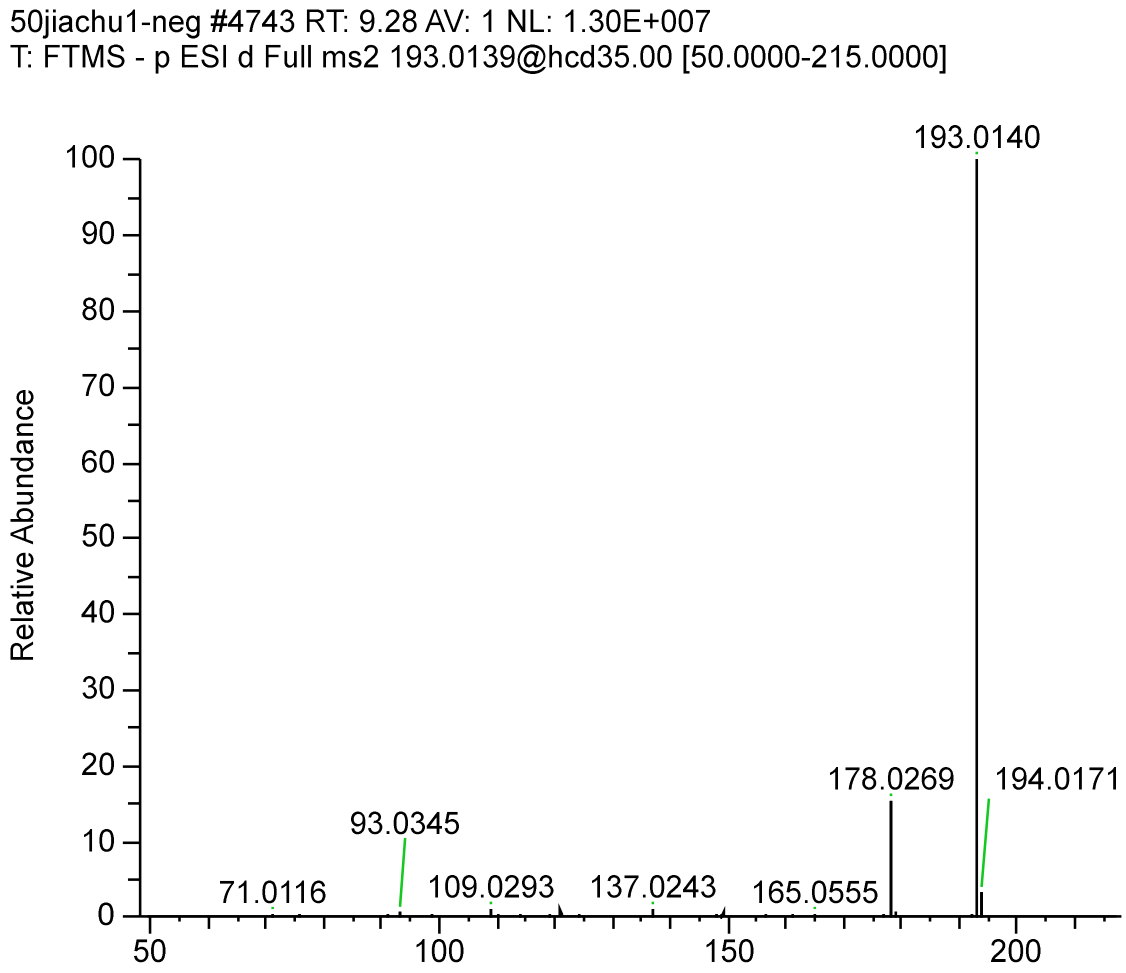

13. Dihydrorobinetin

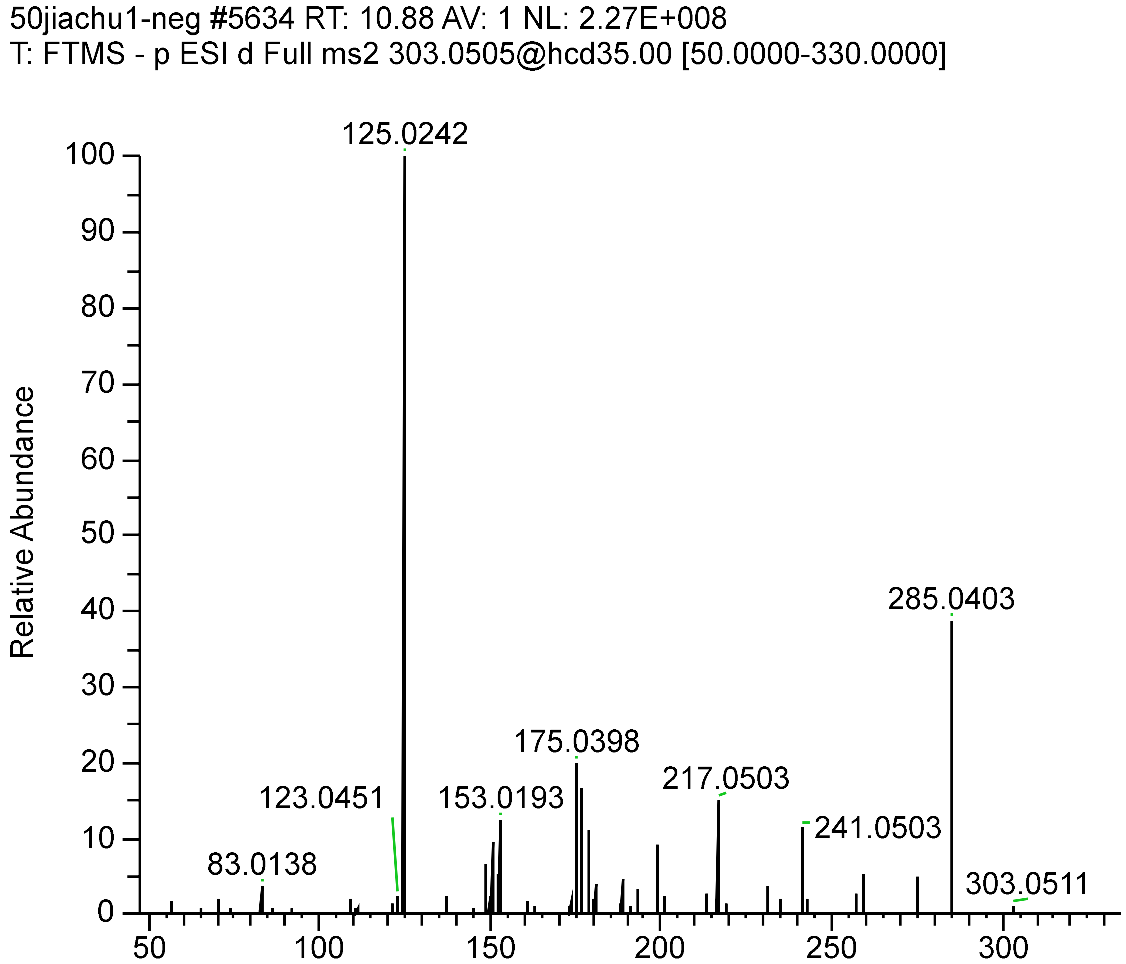

14. Phloroglucinol

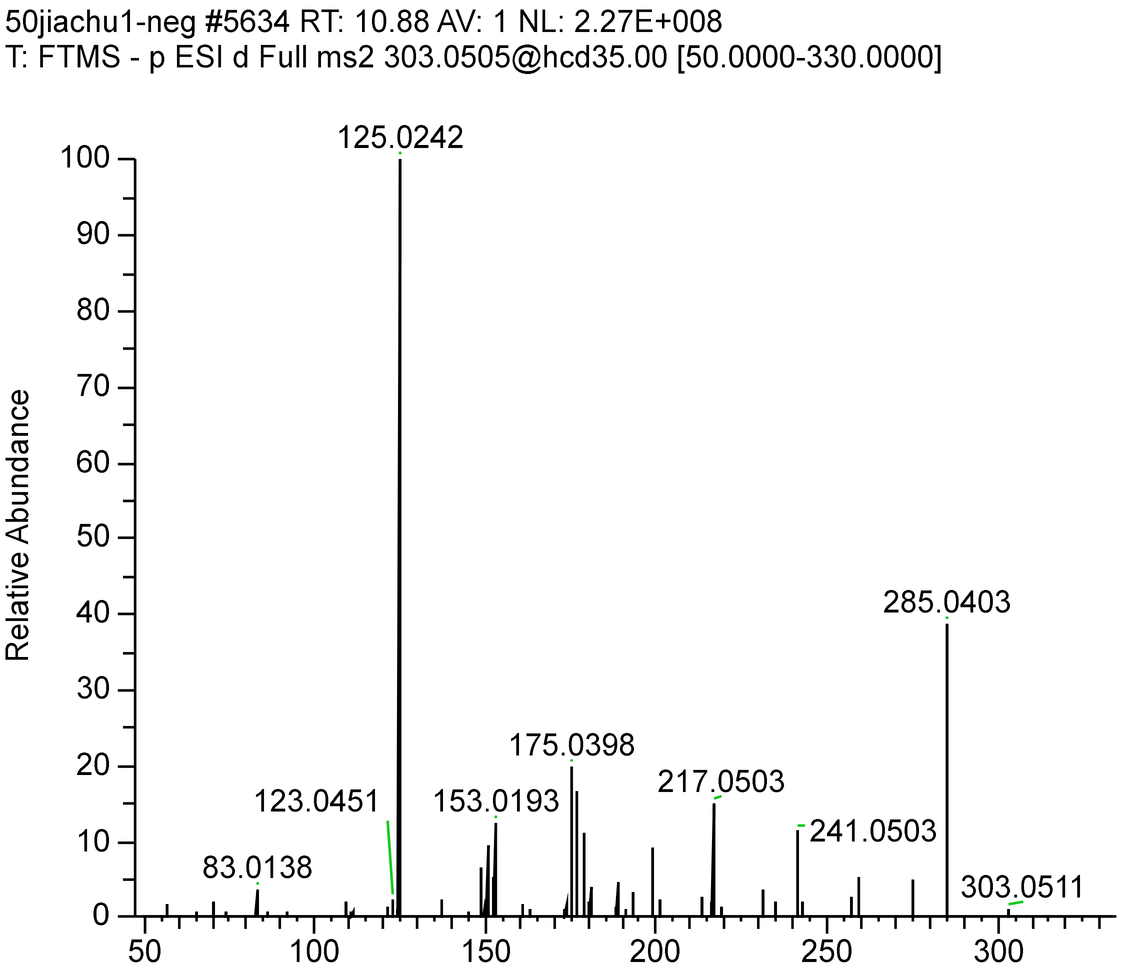

15. D-(+)-Tryptophan

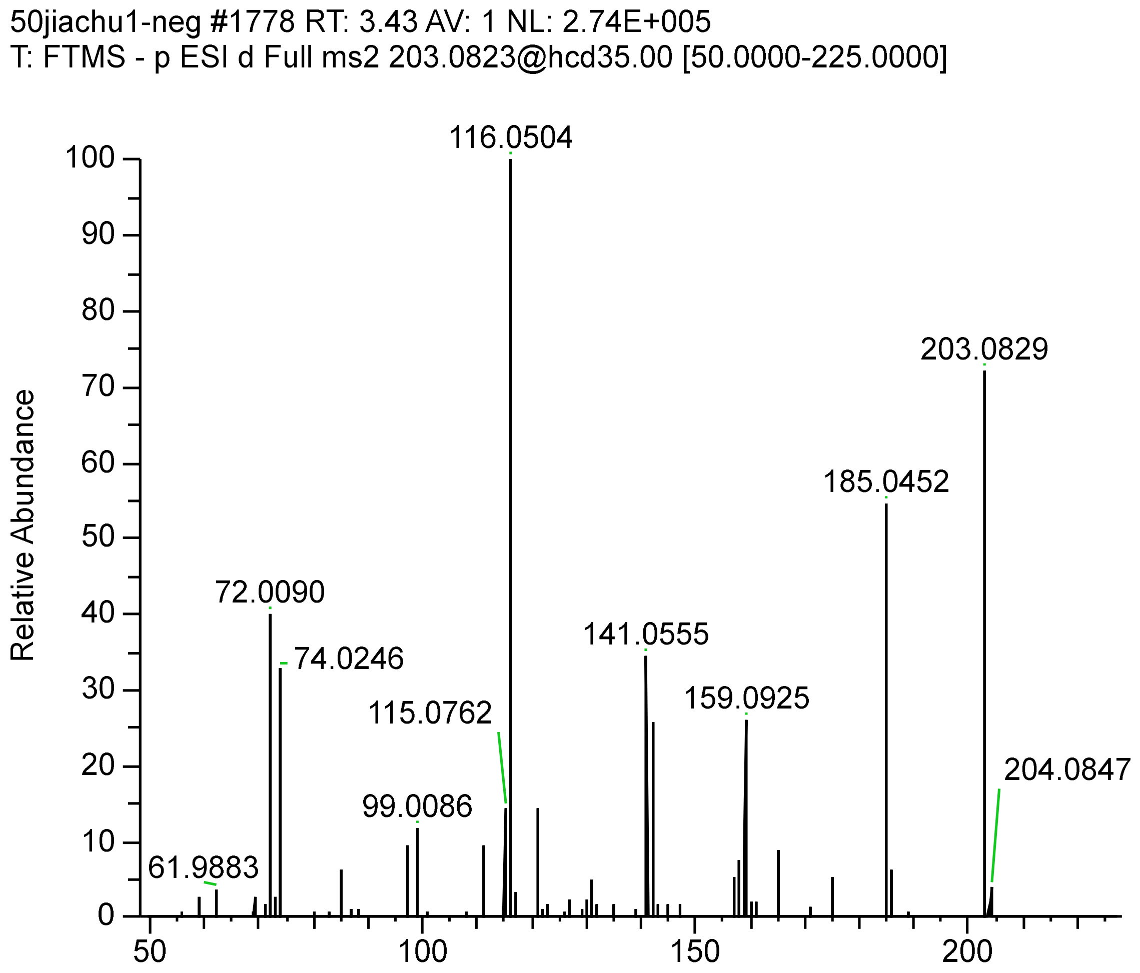

16. Isovitexin

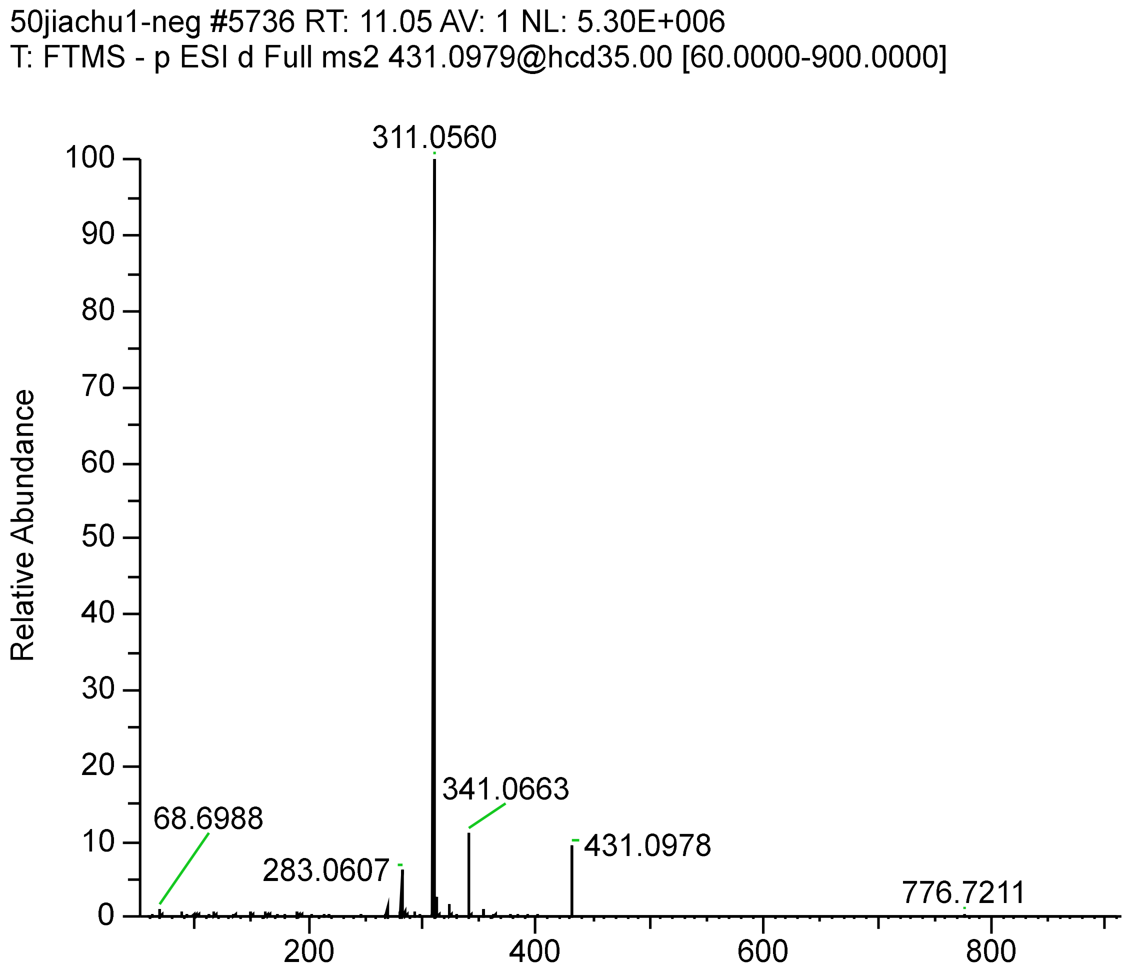

17. Quercetin-3-O-glucoside

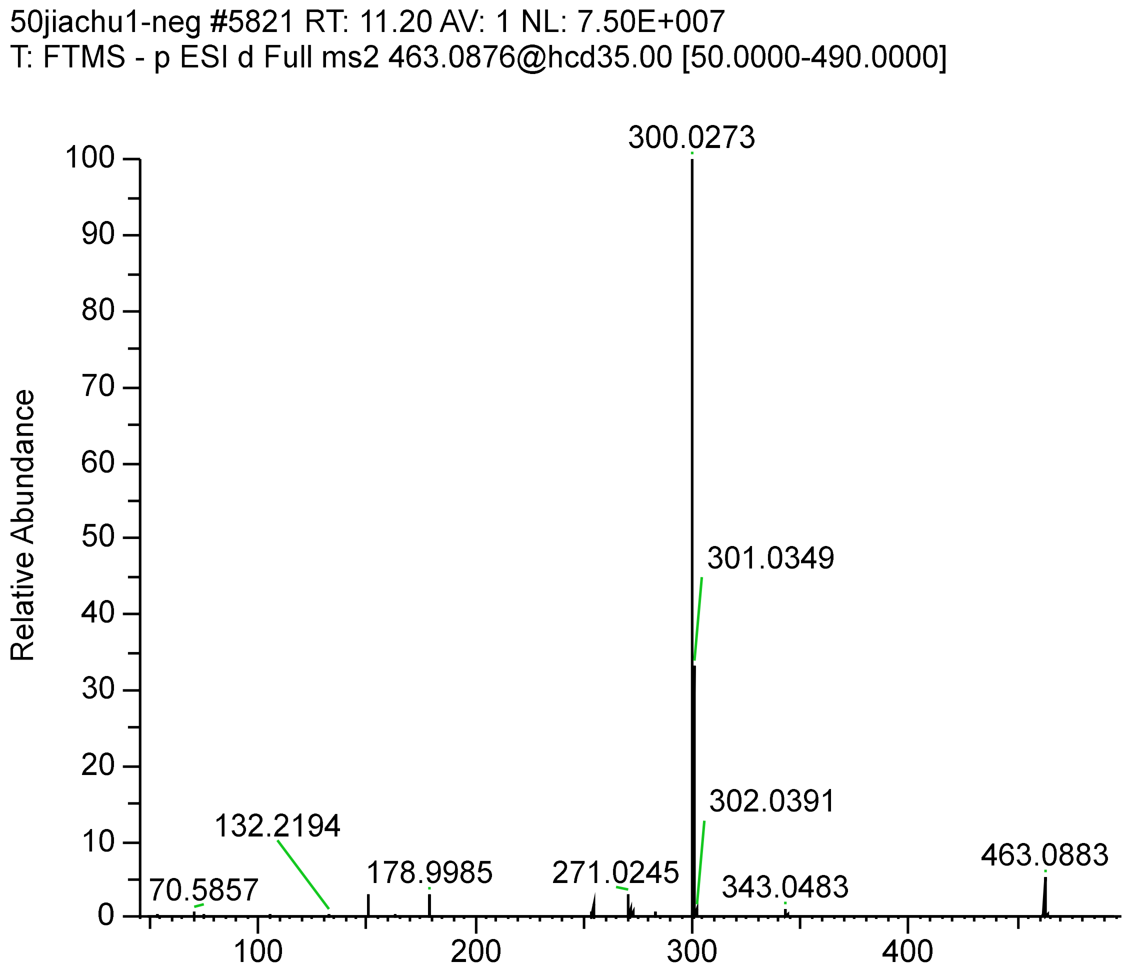

18. trans-Cinnamic acid

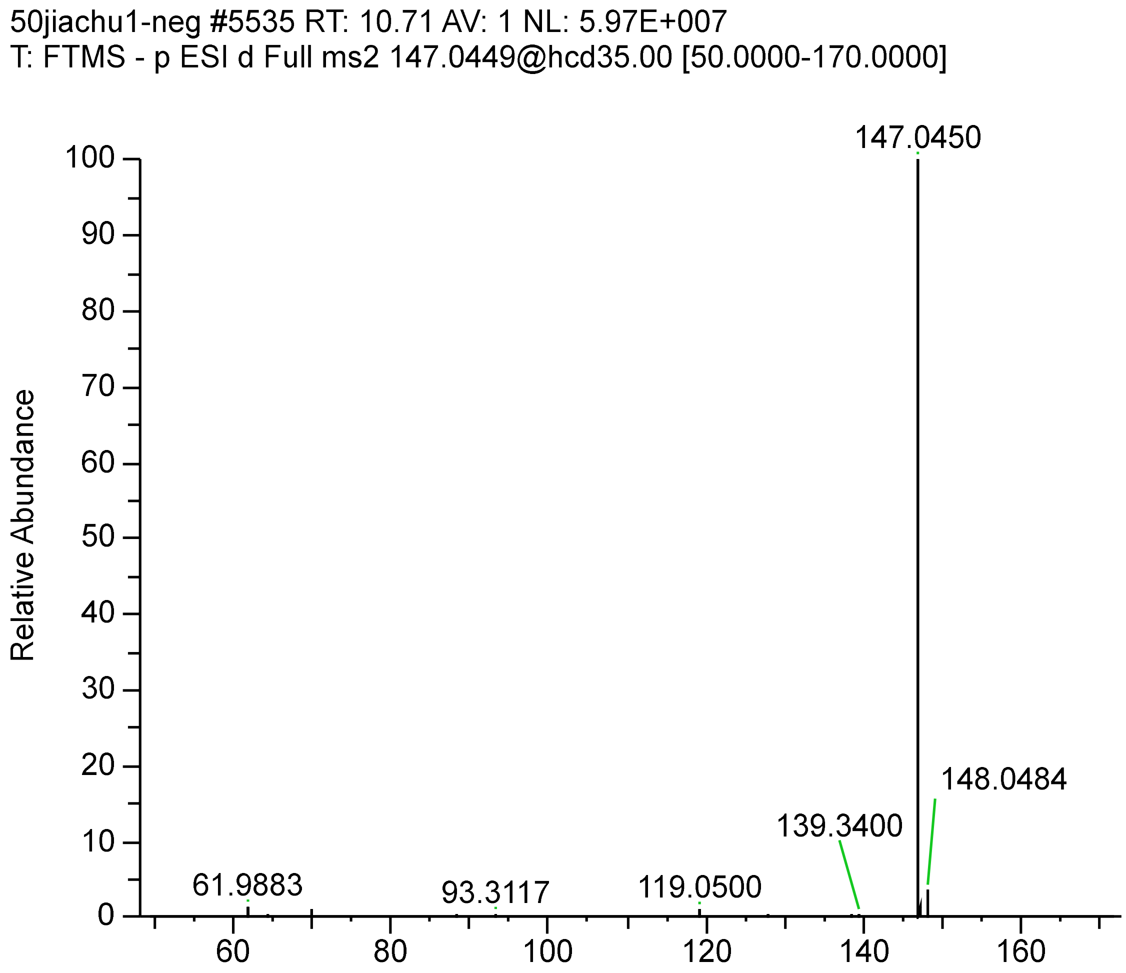

19. Isoorientin

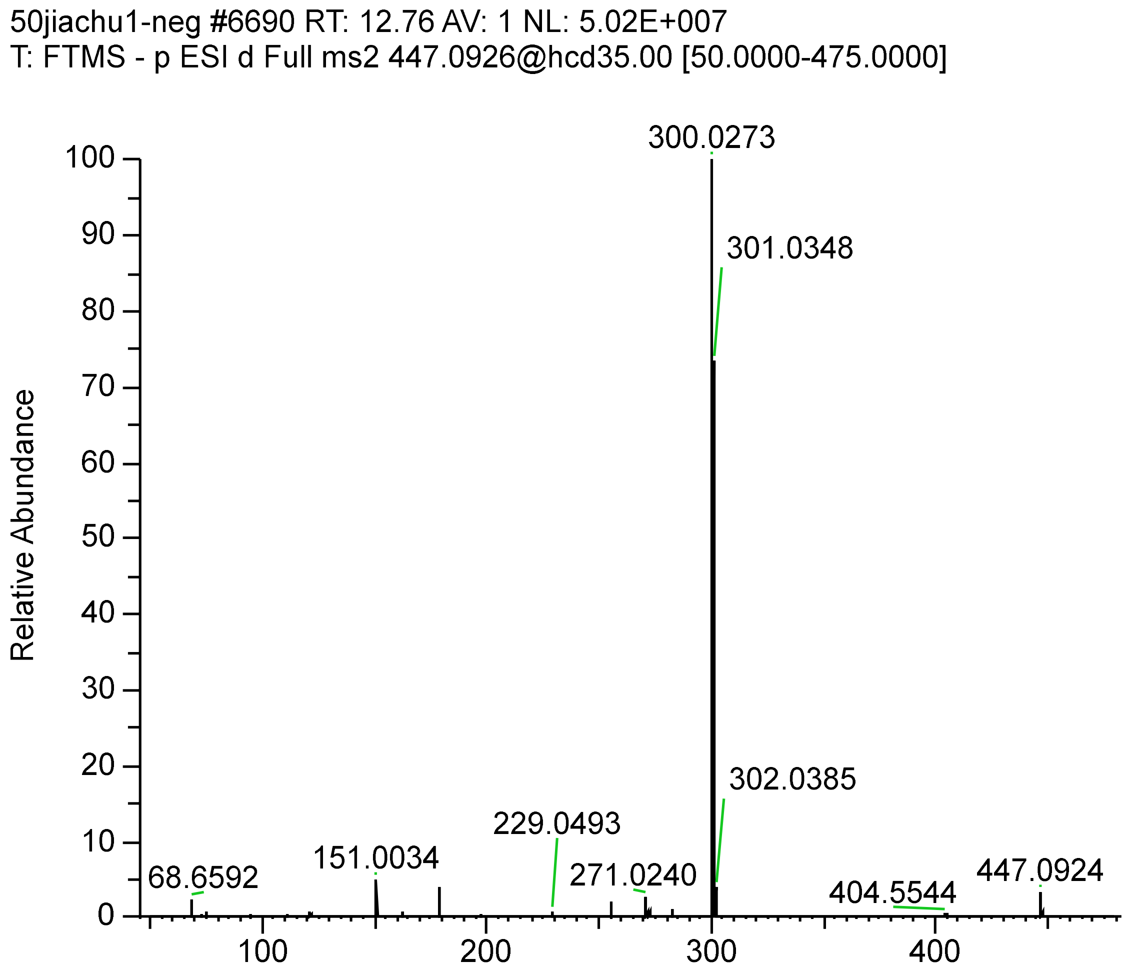

20. Fustin

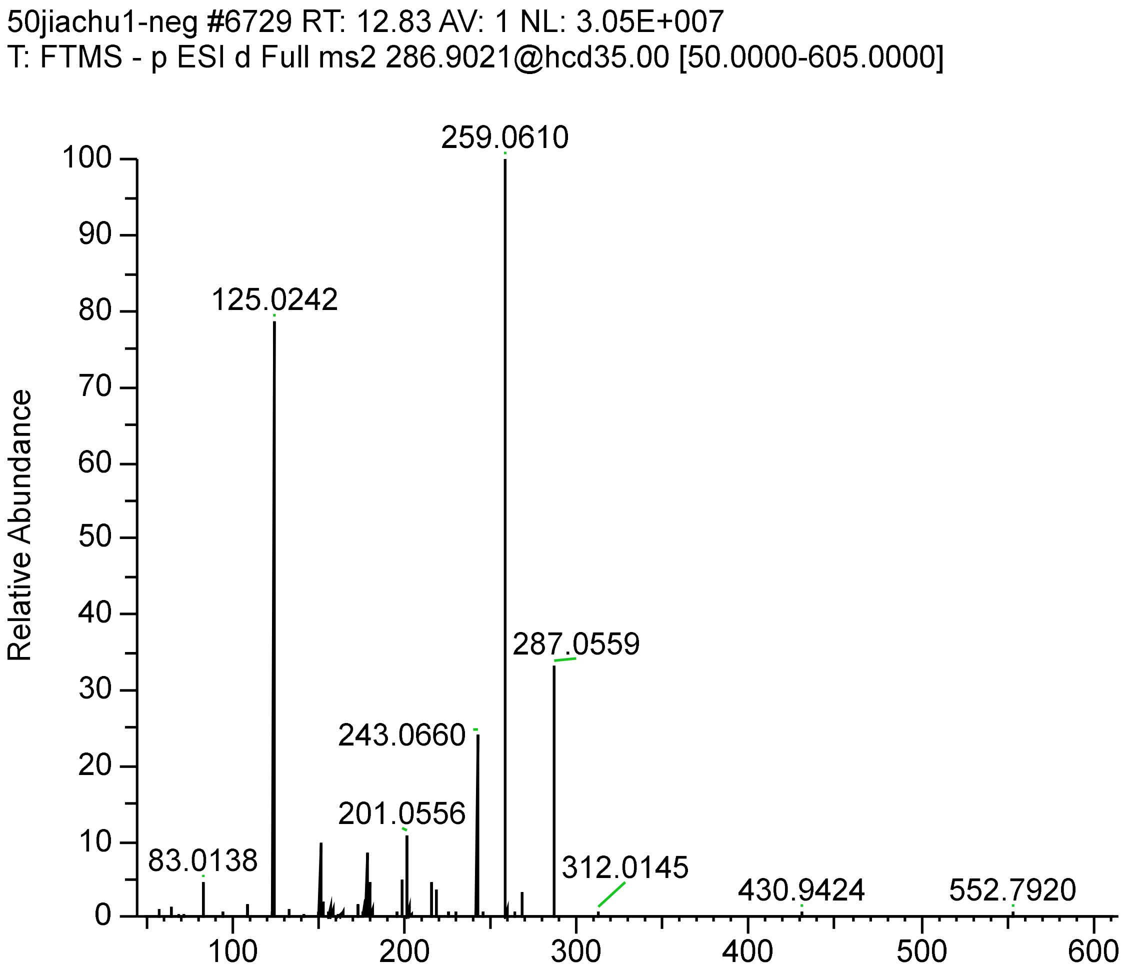
50jiachu1-neg #6729 RT: 12.83 AV: 1 NL: 3.05E+007
T: FTMS - p ESI d Full ms2 286.9021@hcd35.00 [50.0000-605.0000]
Relative Abundance
259.0610
125.0242
287.0559
243.0660
201.0556
83.0138
312.0145
430.9424
552.7920
0
10
20
30
40
50
60
70
80
90
100
100
200
300
400
500
600

21. Hydrocinnamic acid

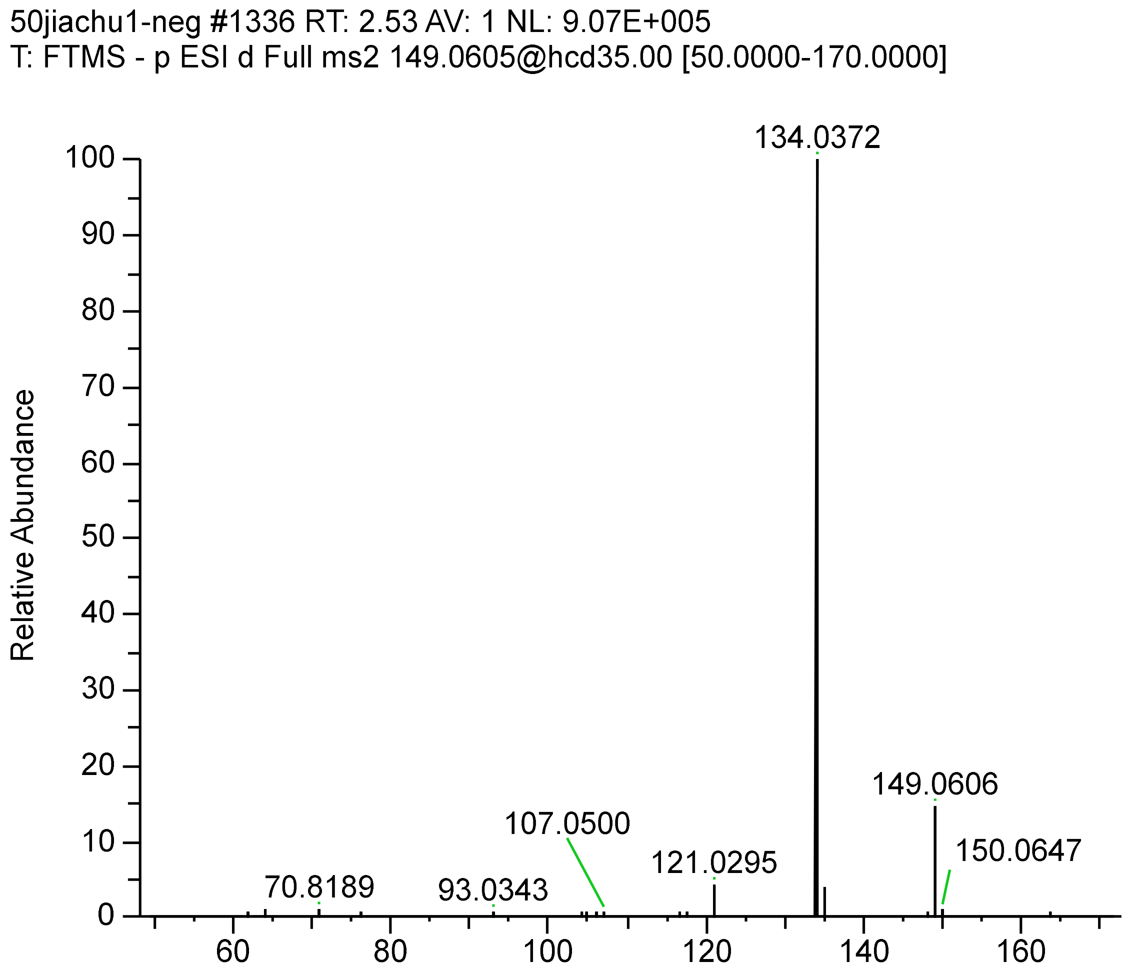

22. Tyramine

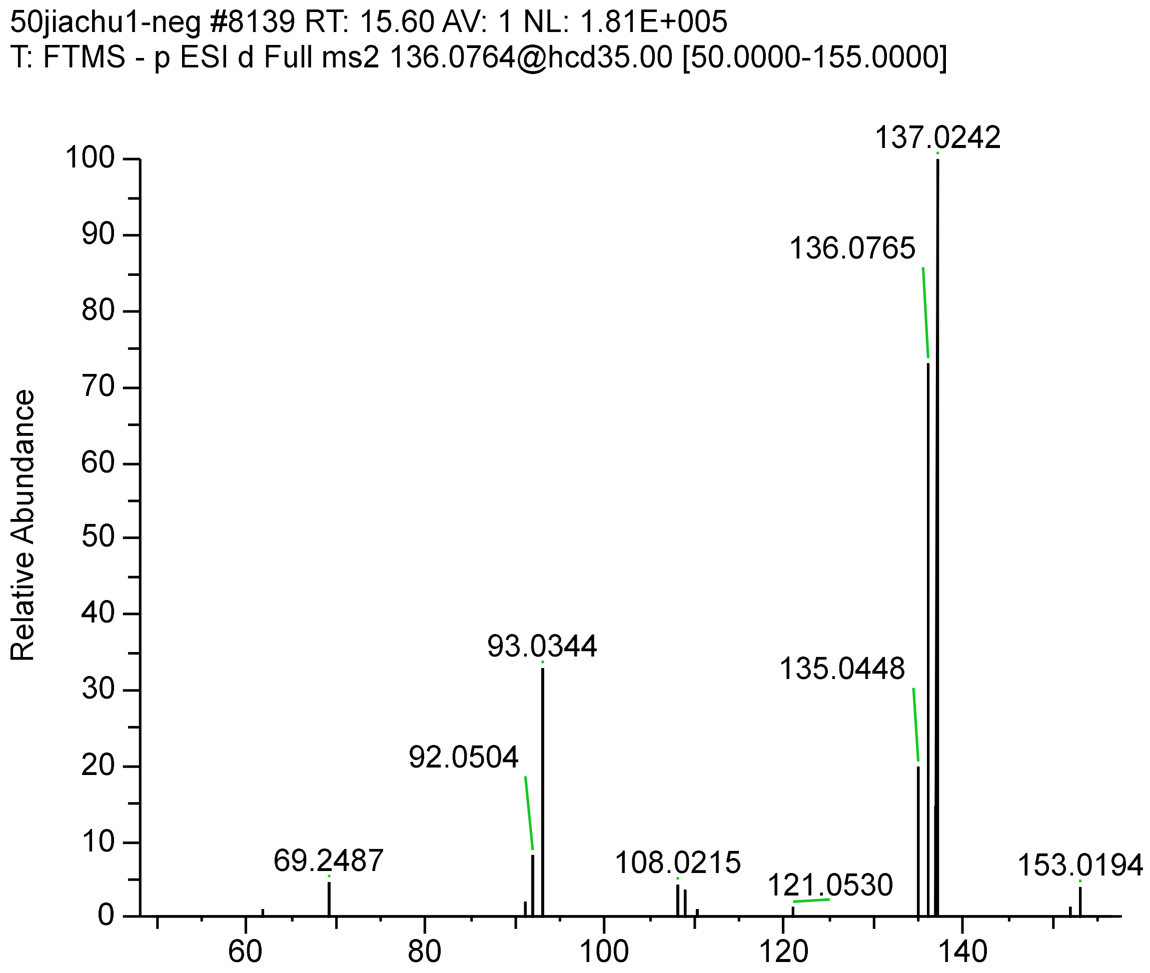
50jiachu1-neg #8139 RT: 15.60 AV: 1 NL: 1.81E+005
T: FTMS - p ESI d Full ms2 136.0764@hcd35.00 [50.0000-155.0000]
Relative Abundance
137.0242
136.0765
93.0344
135.0448
92.0504
69.2487
108.0215
121.0530
153.0194
0
10
20
30
40
50
60
70
80
90
100
60
80
100
120
140

23. Robinetin

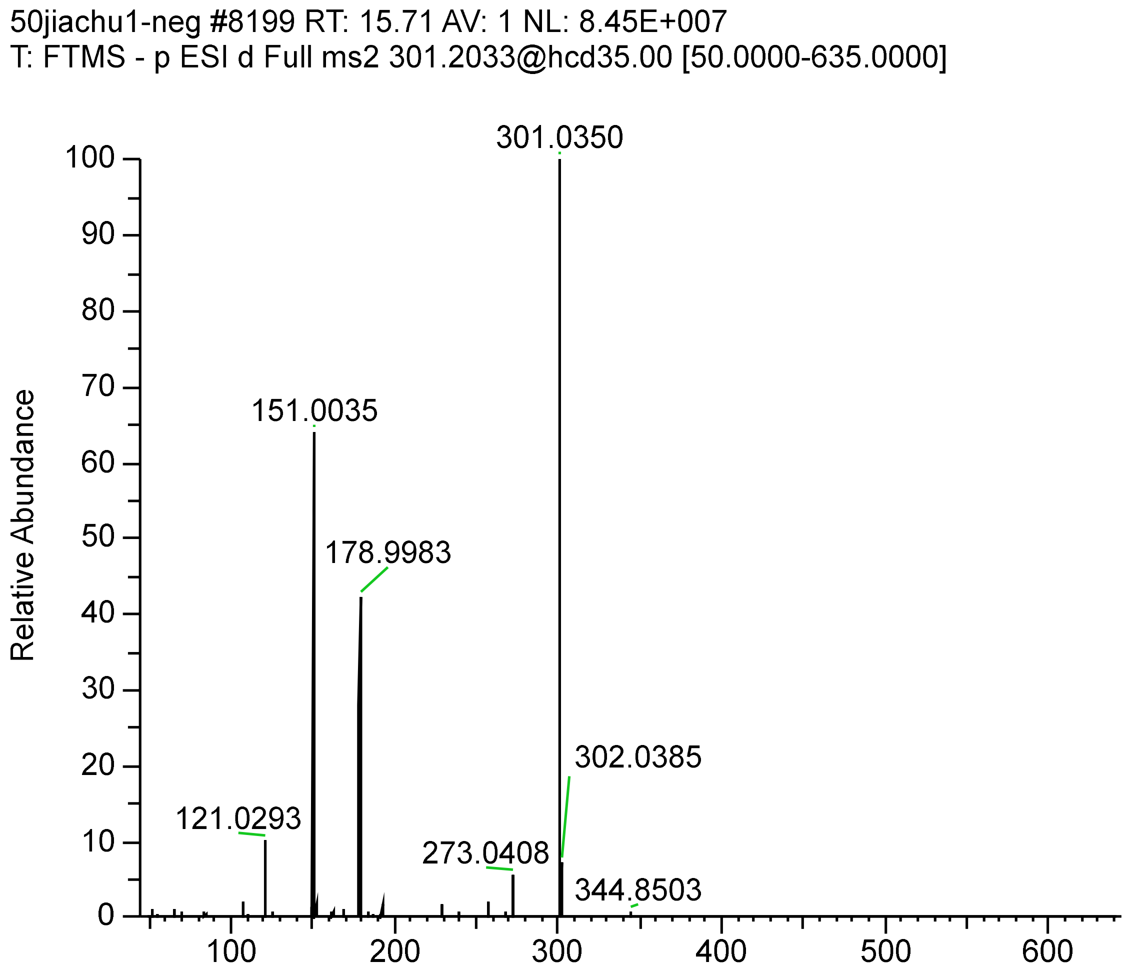

24. Luteolin

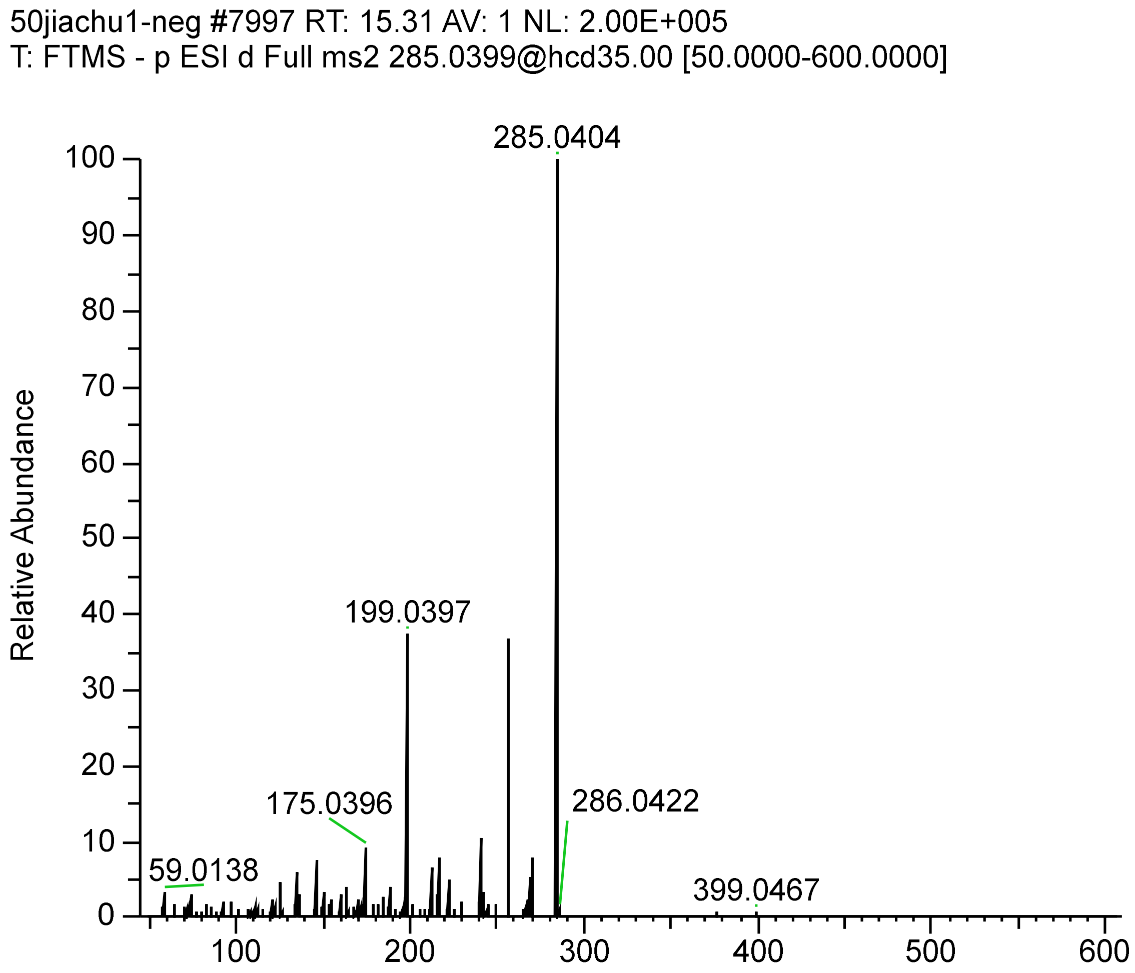
50jiachu1-neg #7997 RT: 15.31 AV: 1 NL: 2.00E+005
T: FTMS - p ESI d Full ms2 285.0399@hcd35.00 [50.0000-600.0000]
Relative Abundance
285.0404
199.0397
175.0396
286.0422
59.0138
399.0467
0
10
20
30
40
50
60
70
80
90
100
100
200
300
400
500
600

25. Azelaic acid

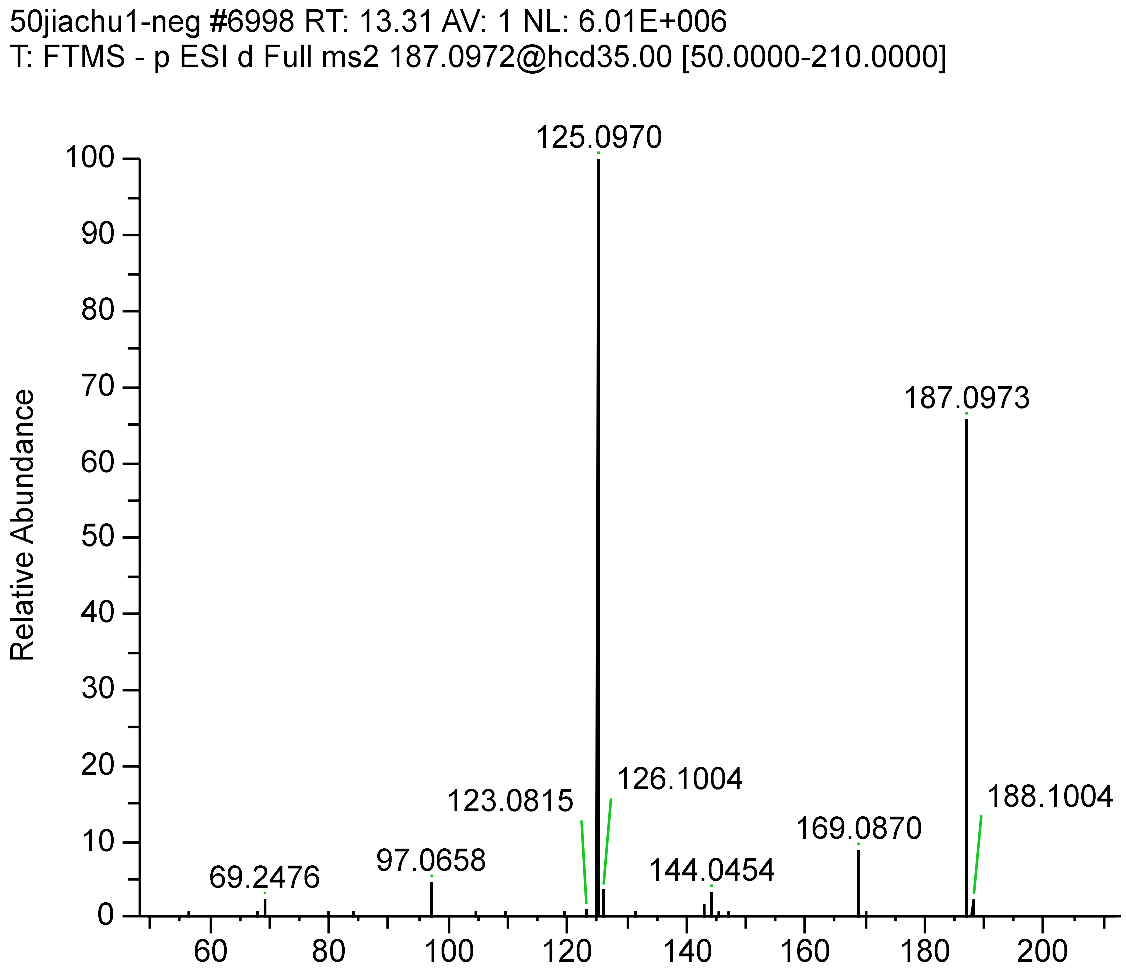

26. Genistein

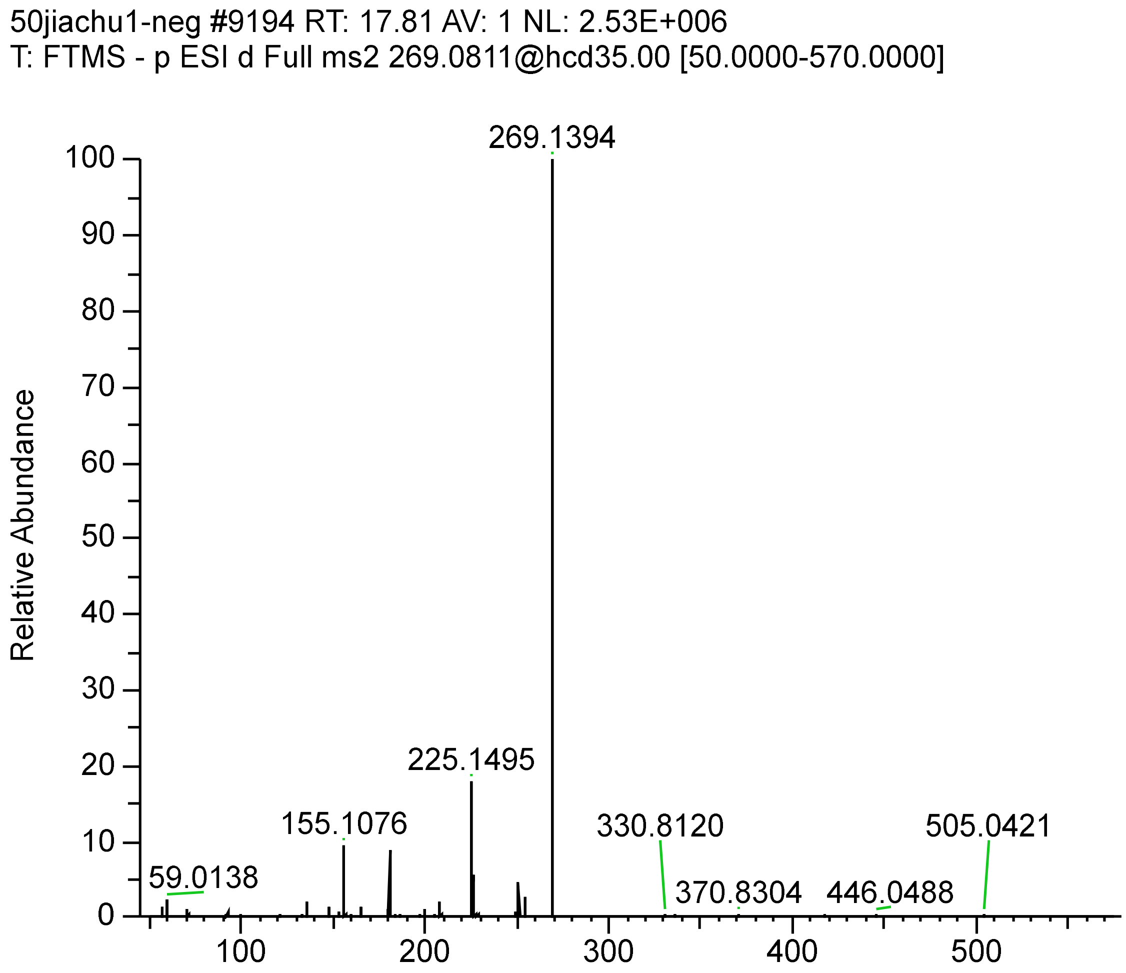

27. Hexadecanedioic acid

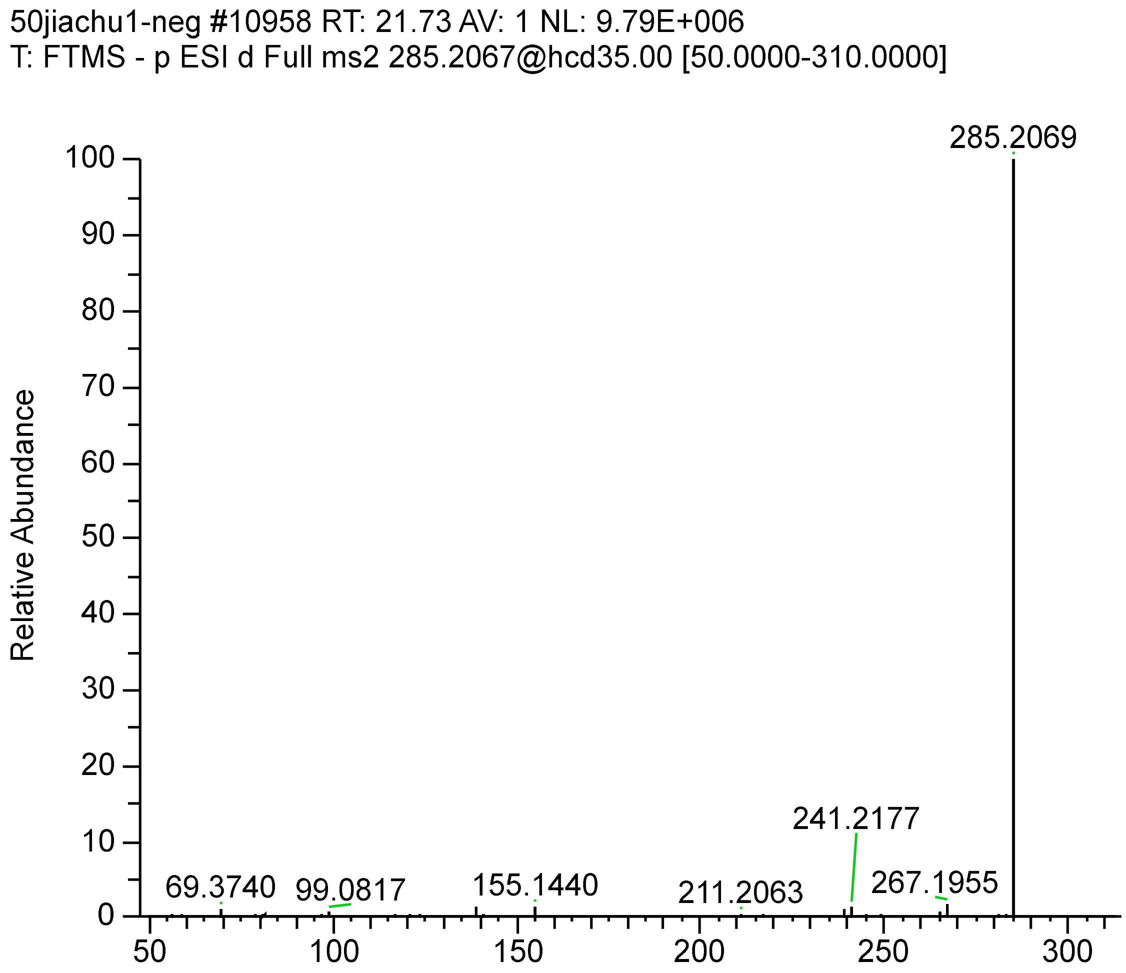

28. 18-β-Glycyrrhetinic acid

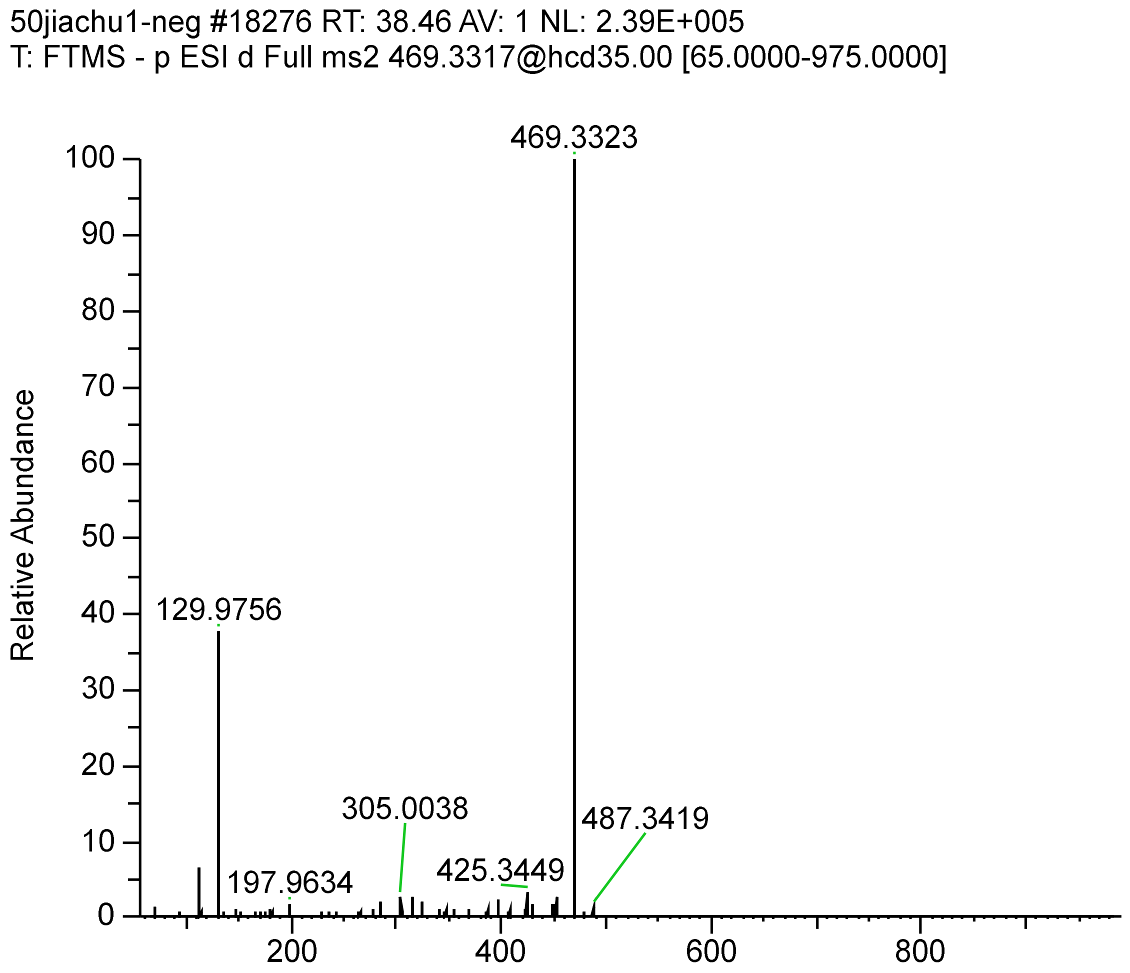

29. Ursolic acid

50jiachu1-neg #19041 RT: 40.21 AV: 1 NL: 5.28E+005
T: FTMS - p ESI d Full ms2 455.3528@hcd35.00 [94.3333-1415.0000]

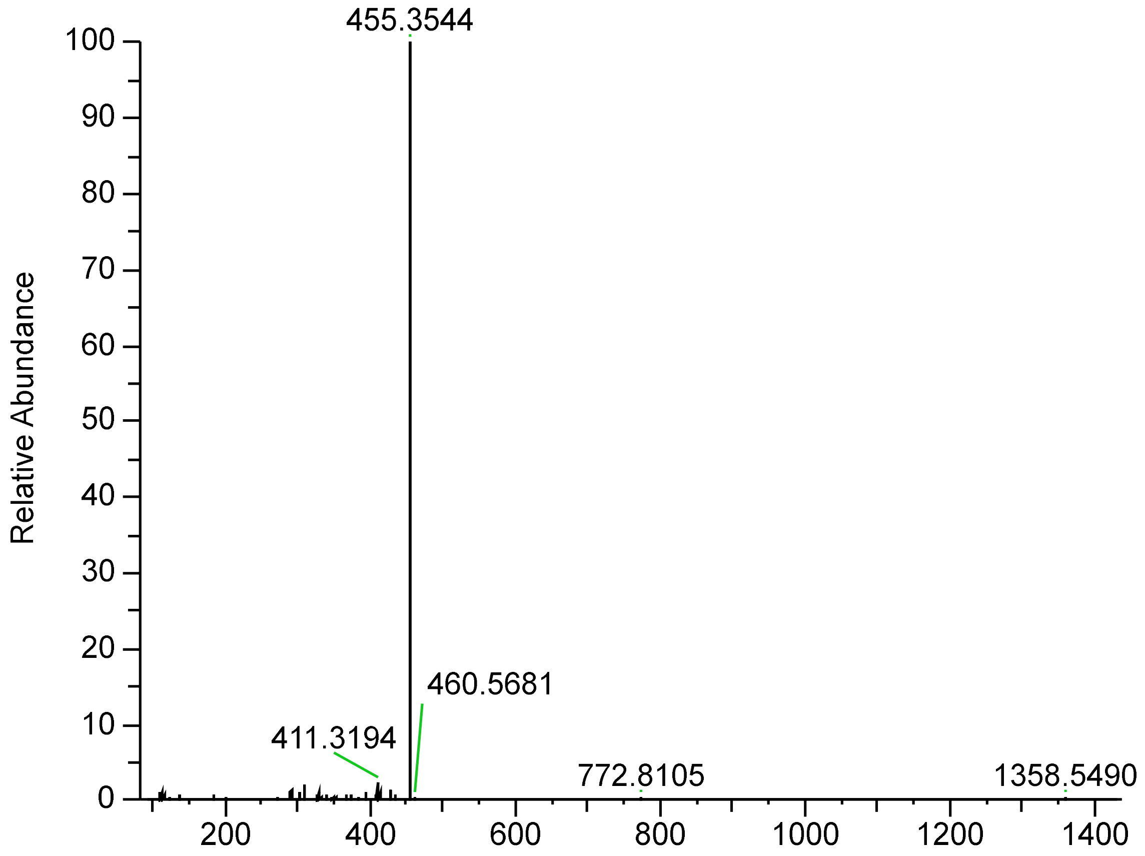

30. Oleanolic acid

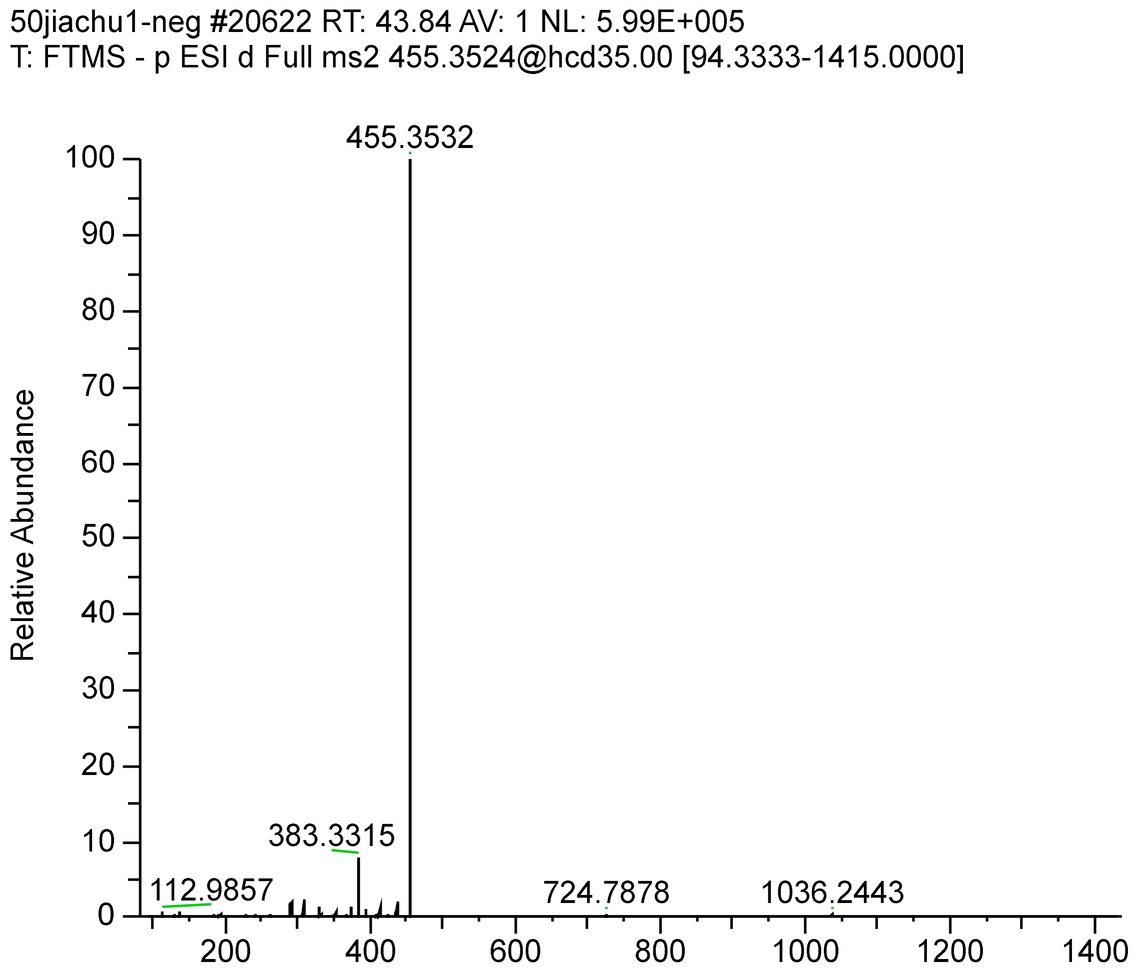

31. L-(+)-Arginine

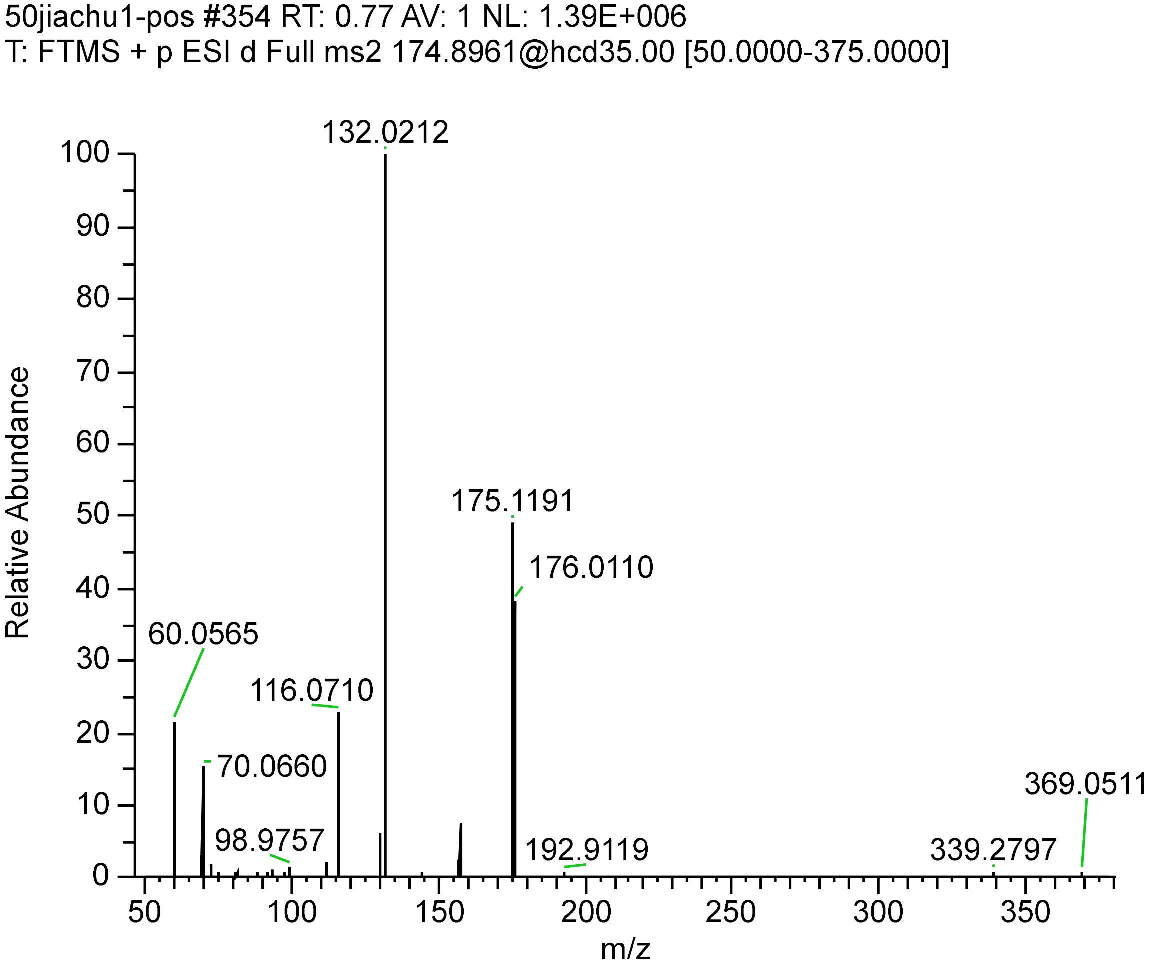

32. L-Tyrosine

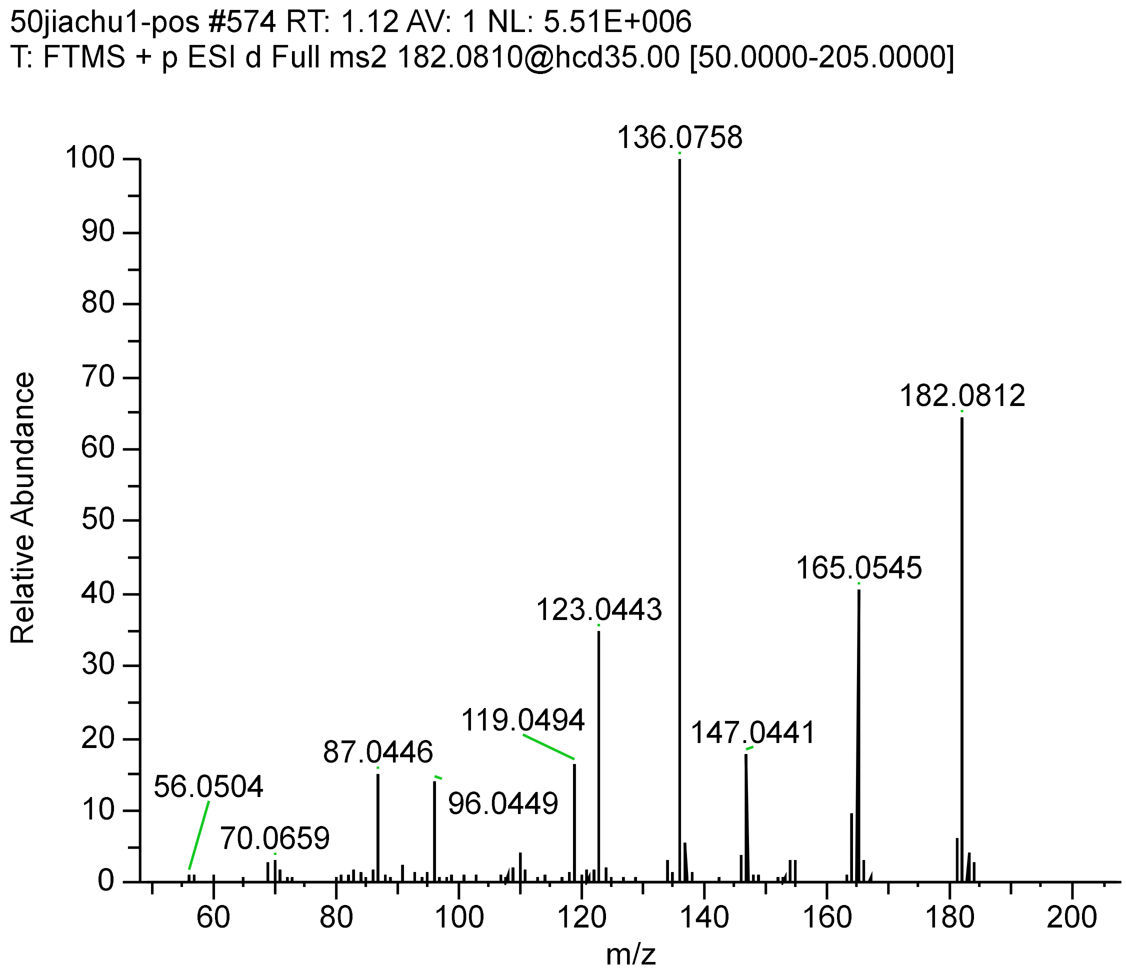

33. Methyl cinnamate

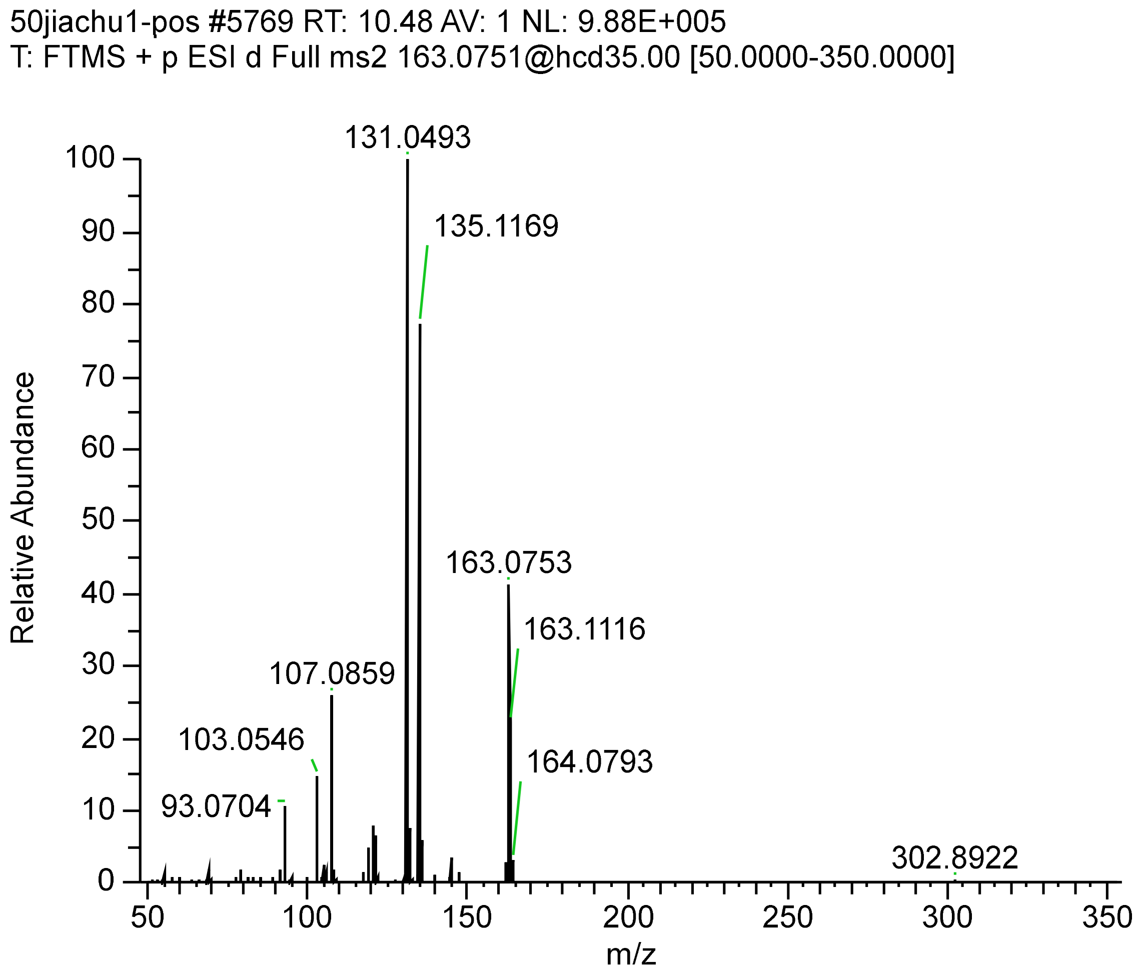

34. 8-Hydroxyquinoline

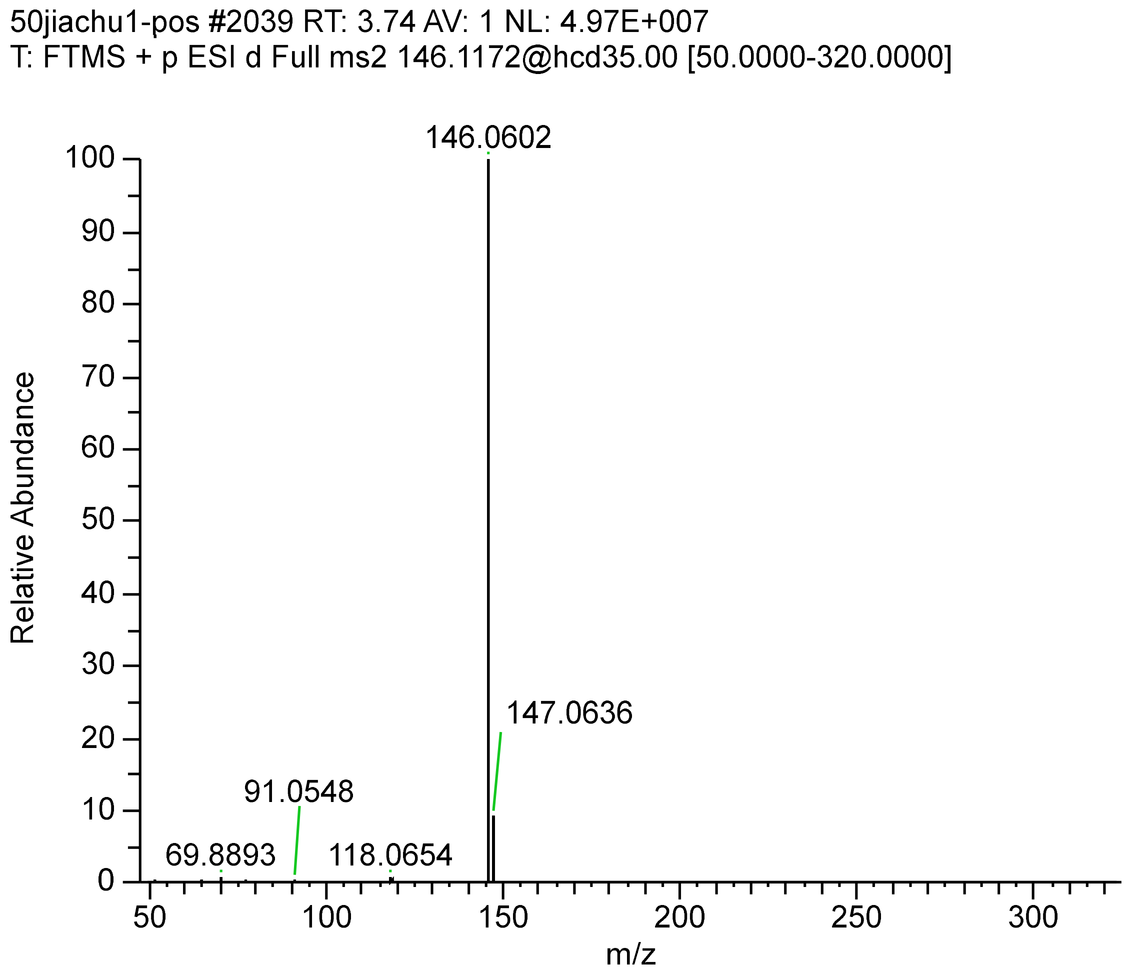

35. Vitexin

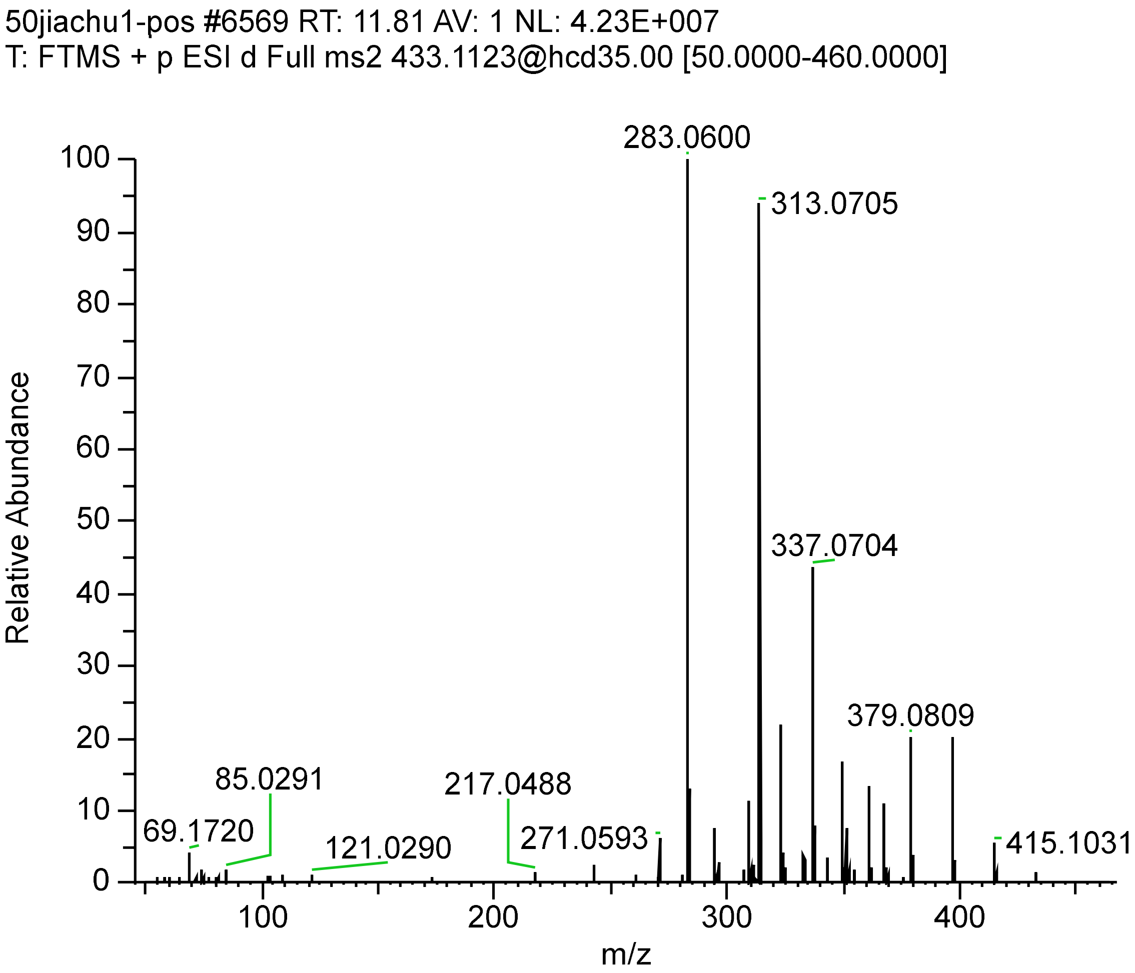

50jiachu1-pos #6569 RT: 11.81 AV: 1 NL: 4.23E+007
T: FTMS + p ESI d Full ms2 433.1123@hcd35.00 [50.0000-460.0000]
283.0600
313.0705
337.0704
379.0809
415.1031
271.0593
217.0488
121.0290
85.0291
69.1720
Relative Abundance
m/z

36. Citral

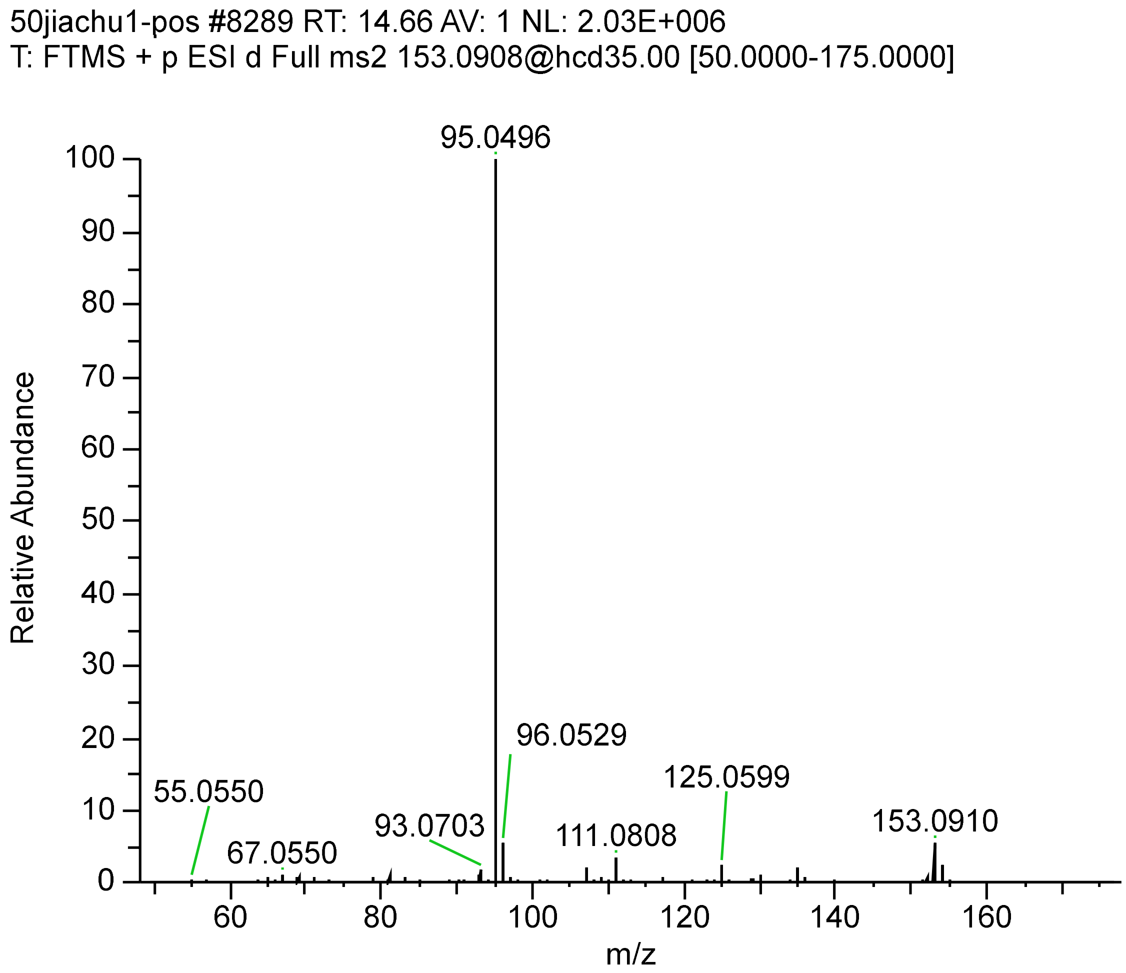
50jiachu1-pos #8289 RT: 14.66 AV: 1 NL: 2.03E+006
T: FTMS + p ESI d Full ms2 153.0908@hcd35.00 [50.0000-175.0000]
95.0496
96.0529
125.0599
55.0550
153.0910
93.0703
111.0808
67.0550
Relative Abundance
m/z
0
10
20
30
40
50
60
70
80
90
100
60
80
100
120
140
160

37. Berberine

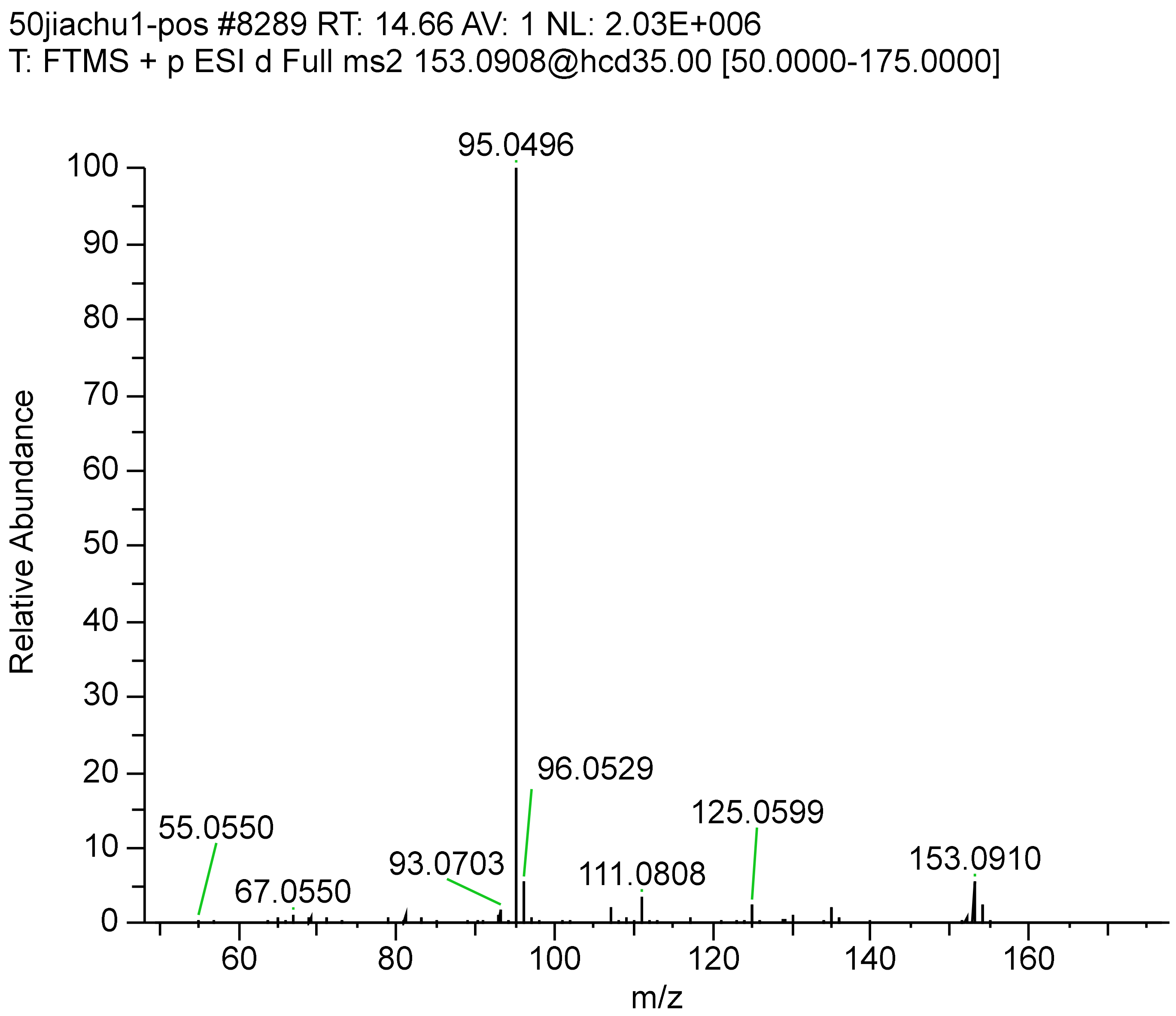

38. Rhamnetin

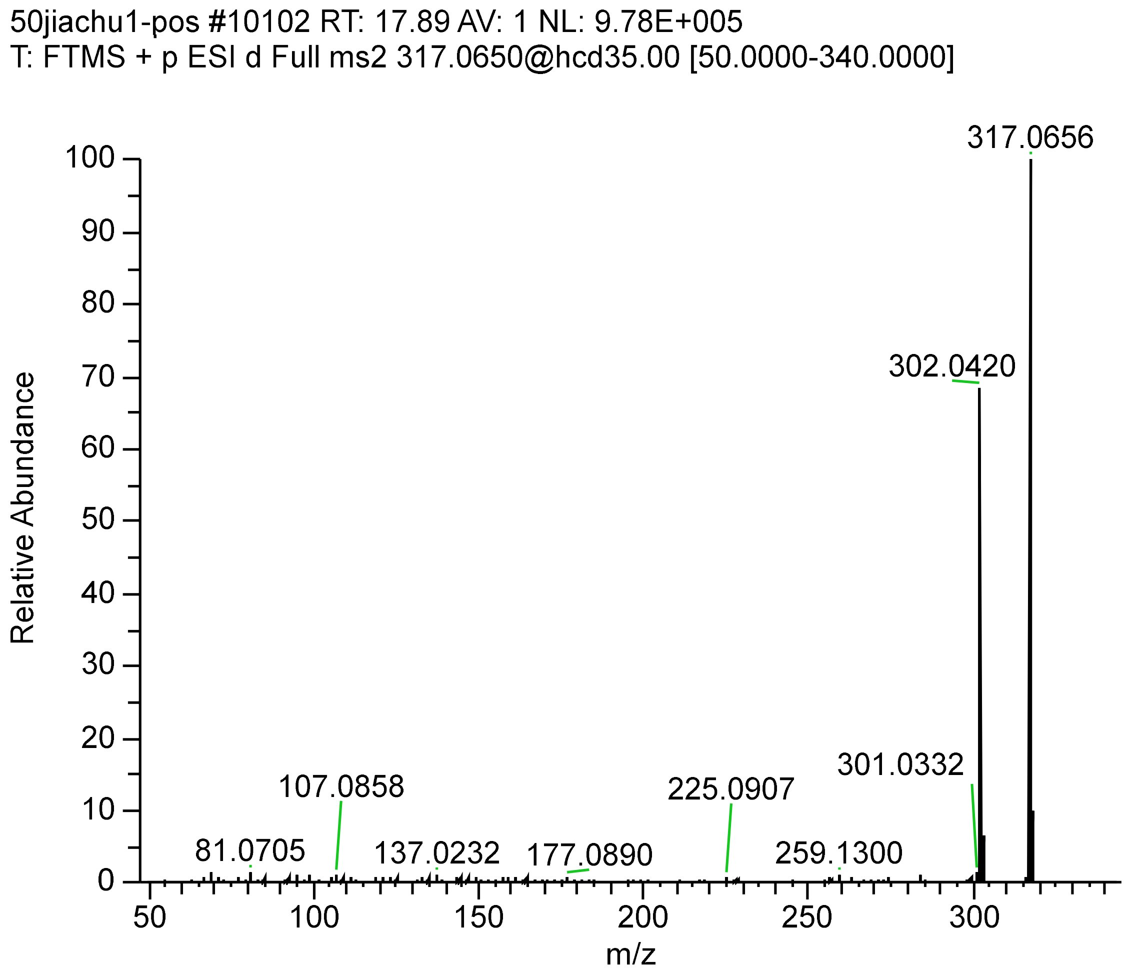

39. Stigmastane-3,6-dione

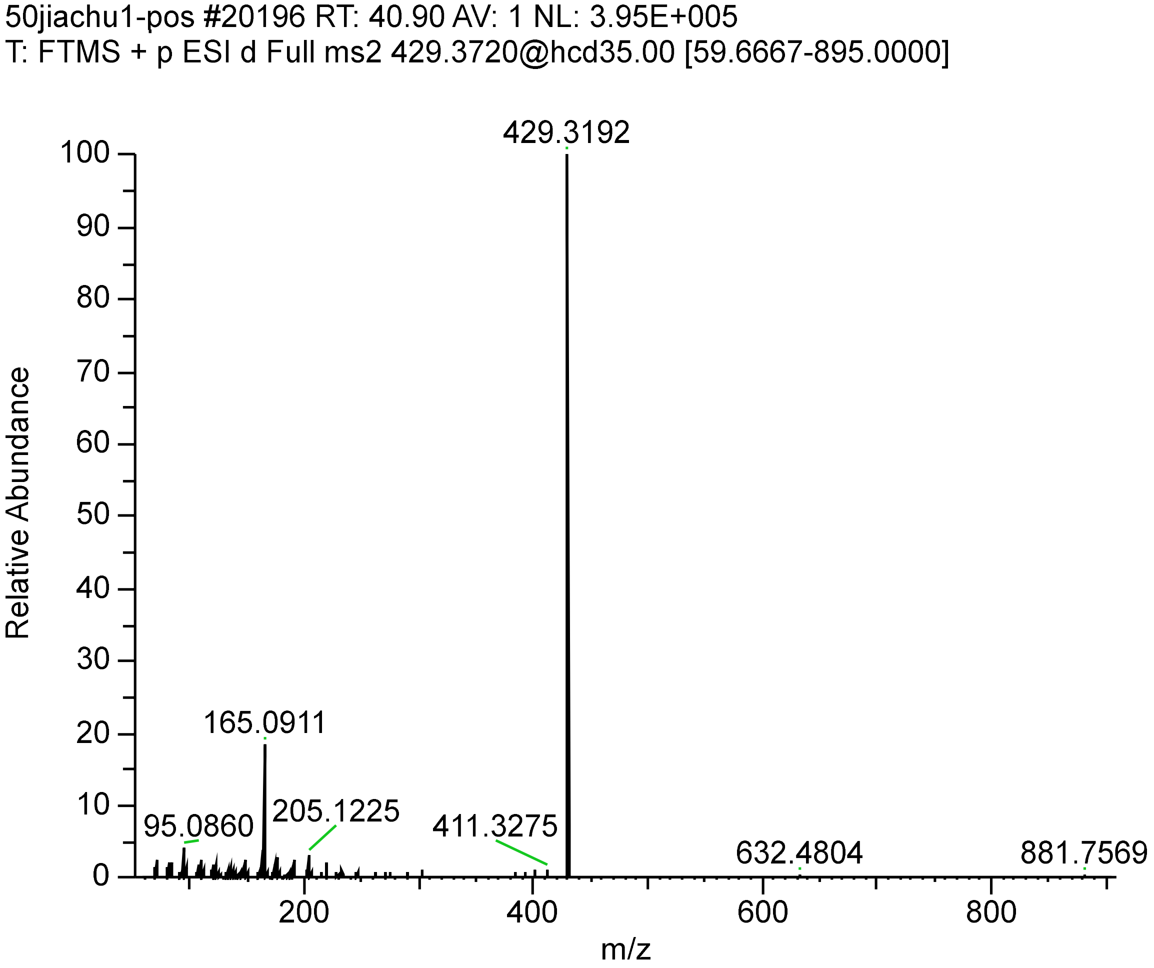

Supplement: Supplementary Materials — Supplementary material 1: the mass spectra of 39 identified compounds was shown in supplementary material 1. Through the mass spectrum of each component, the structure of the compound could be speculated, and the type of compounds was indirectly determined. [file 5988310.f1.pdf]
